# Mutation impact on mRNA versus protein expression across human cancers

**DOI:** 10.1093/gigascience/giae113

**Published:** 2025-01-06

**Authors:** Yuqi Liu, Abdulkadir Elmas, Kuan-lin Huang

**Affiliations:** Department of Genetics and Genomic Sciences, Department of Artificial Intelligence and Human Health, Center for Transformative Disease Modeling, Tisch Cancer Institute, Icahn Genomics Institute, Icahn School of Medicine at Mount Sinai, New York, NY 10029, USA; Department of Genetics and Genomic Sciences, Department of Artificial Intelligence and Human Health, Center for Transformative Disease Modeling, Tisch Cancer Institute, Icahn Genomics Institute, Icahn School of Medicine at Mount Sinai, New York, NY 10029, USA; Department of Genetics and Genomic Sciences, Department of Artificial Intelligence and Human Health, Center for Transformative Disease Modeling, Tisch Cancer Institute, Icahn Genomics Institute, Icahn School of Medicine at Mount Sinai, New York, NY 10029, USA

**Keywords:** Proteogenomics, cancer mutations, protein expression, quantitative trait loci (QTL), variant effects

## Abstract

**Background:**

Cancer mutations are often assumed to alter proteins, thus promoting tumorigenesis. However, how mutations affect protein expression—in addition to gene expression—has rarely been systematically investigated. This is significant as mRNA and protein levels frequently show only moderate correlation, driven by factors such as translation efficiency and protein degradation. Proteogenomic datasets from large tumor cohorts provide an opportunity to systematically analyze the effects of somatic mutations on mRNA and protein abundance and identify mutations with distinct impacts on these molecular levels.

**Results:**

We conduct a comprehensive analysis of mutation impacts on mRNA- and protein-level expressions of 953 cancer cases with paired genomics and global proteomic profiling across 6 cancer types. Protein-level impacts are validated for 47.2% of the somatic expression quantitative trait loci (seQTLs), including *CDH1* and *MSH3* truncations, as well as other mutations from likely “long-tail” driver genes. Devising a statistical pipeline for identifying somatic protein-specific QTLs (spsQTLs), we reveal several gene mutations, including *NF1* and *MAP2K4* truncations and *TP53* missenses showing disproportional influence on protein abundance not readily explained by transcriptomics. Cross-validating with data from massively parallel assays of variant effects (MAVE), *TP53* missenses associated with high tumor TP53 proteins are more likely to be experimentally confirmed as functional.

**Conclusion:**

This study reveals that somatic mutations can exhibit distinct impacts on mRNA and protein levels, underscoring the necessity of integrating proteogenomic data to comprehensively identify functionally significant cancer mutations. These insights provide a framework for prioritizing mutations for further functional validation and therapeutic targeting.

## Introduction

Cancer arises from the acquisition of mutations that confer selective advantages. Most of these mutations are thought to affect cellular functions by regulating the expression of gene products. For example, truncations can result in nonsense-mediated decay (NMD) [1, 2], which protects eukaryotic cells through degrading premature termination codon (PTC) bearing mRNA [3]. Additionally, a fraction of cancer mutations may uniquely affect protein abundance but not mRNA expression. However, previous studies characterizing genomic mutations affecting mRNA versus protein levels have focused on germline variants as expression quantitative trait loci (eQTL) [4–6]. While other cancer studies have characterized the effect of somatic mutations on mRNA expression levels [7–9], it remains unclear how somatic mutations may affect protein abundance. The gap of knowledge is critical given that mRNA and protein levels are only moderately correlated [10–13]. A myriad of factors, including cell state transition, signal delay, translation on demand, and cellular energy constraint, can lead to discrepancies between mRNA and protein levels [14]. Understanding protein-level consequences of cancer mutations is critical in identifying functionally important mutations and revealing their downstream mechanisms.

In recent years, advances in mass spectrometry (MS) technologies have generated a wealth of global proteomic profiles of primary tumor cohorts, many of which also have concurrent genomic and transcriptomic profiling [15–20]. These proteogenomic datasets present ample opportunities to validate somatic mutations that show concordant impacts on downstream mRNA and protein levels. On the other hand, protein abundance may also be uniquely influenced by the efficiency of protein translation, transport, and degradation. Thus, proteogenomic analyses can reveal mutations that disproportionally impact protein abundances that may not be found using genomic analyses alone.

Herein, we conducted a systematic analysis to decode the relationship between somatic mutations versus mRNA and protein levels using data from nearly a thousand cases across 6 cancer types in prospective and retrospective cohorts from the Clinical Proteomic Tumor Analysis Consortium (CPTAC). We identified mutations showing concordant effects at both mRNA and protein expression levels *in cis*, as well as those that showed protein-specific effects. We further examined how mutations associated with expression changes may predict *in vitro* and *in vivo* functional effects measured by a massively parallel assay of variant effects (MAVE) of TP53 [21]. Our results highlight the importance of pairing genomic and proteomic analyses to prioritize functionally important mutations.

## Results

### Mutation impacts on the mRNA and protein levels

**Figure 1:**
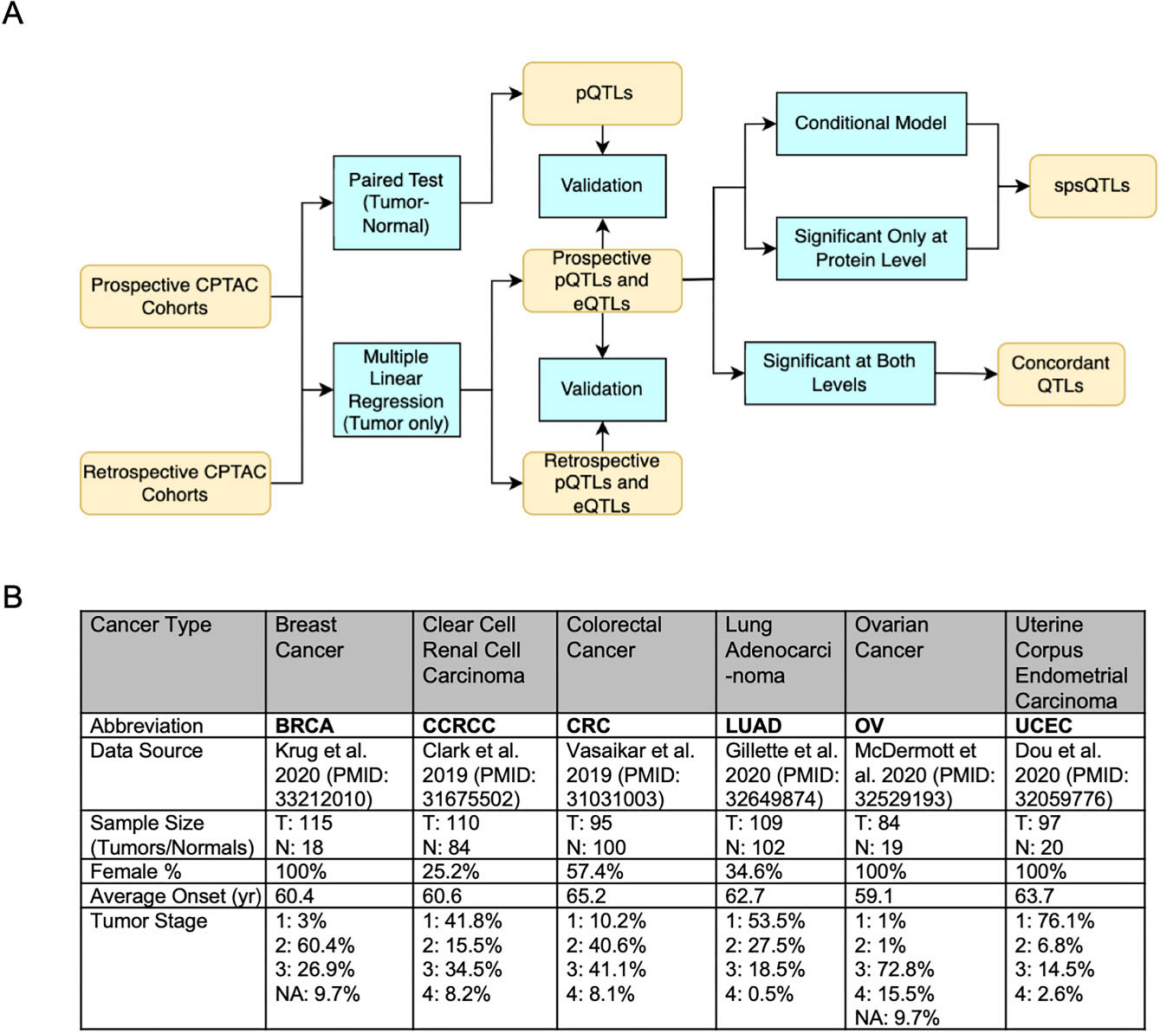
Overview of the study workflow and proteogenomic cohorts. (A) Study workflow to identify eQTLs, pQTLs, concordant QTLs (between mRNA and protein levels), and spsQTLs showing disproportional effects on protein expression. (B) Summary of the prospective CPTAC proteogenomic cohorts used for the discovery analyses, including cancer type abbreviation, data source, sample size of tumor (T) and normal (N) tissues, female percentage, average onset age in years, and tumor stage distribution.

Following the study workflow (Fig. 1A), we first sought to identify somatic mutations that may impact the corresponding gene’s mRNA expression (somatic eQTL, termed seQTL below) and protein abundance (somatic pQTL, termed spQTL below) in primary tumor tissue samples. We performed a multiple regression analysis adjusted for age, gender, ethnicity, and Tandem Mass Tag (TMT) [22] batch using the prospective CPTAC datasets that included matched DNA sequencing (DNA-seq), RNA sequencing (RNA-seq), and MS global proteomics data of primary tumor samples across 6 cancer types (see Methods, Fig. 1B), including 115 breast cancer (BRCA) [19], 95 colorectal cancer (CRC) [16], 110 clear cell renal cell carcinoma (CCRCC) [15], 109 lung adenocarcinoma (LUAD) [17], 84 ovarian cancer (OV) [20], and 97 uterine corpus endometrial carcinoma (UCEC) [18] samples, as well as proteogenomic datasets for additional, retrospective BRCA [11], CRC [13], and OV [12] cohorts from CPTAC for validation (Supplementary Fig. S1A). We focused on coding mutations given the coverage of the whole-exome sequencing (WES) data used in CPTAC studies; the analyses were further stratified for truncations, missense, and synonymous mutations given their likely different mechanisms of action in affecting levels of the mutated gene product.

**Figure 2:**
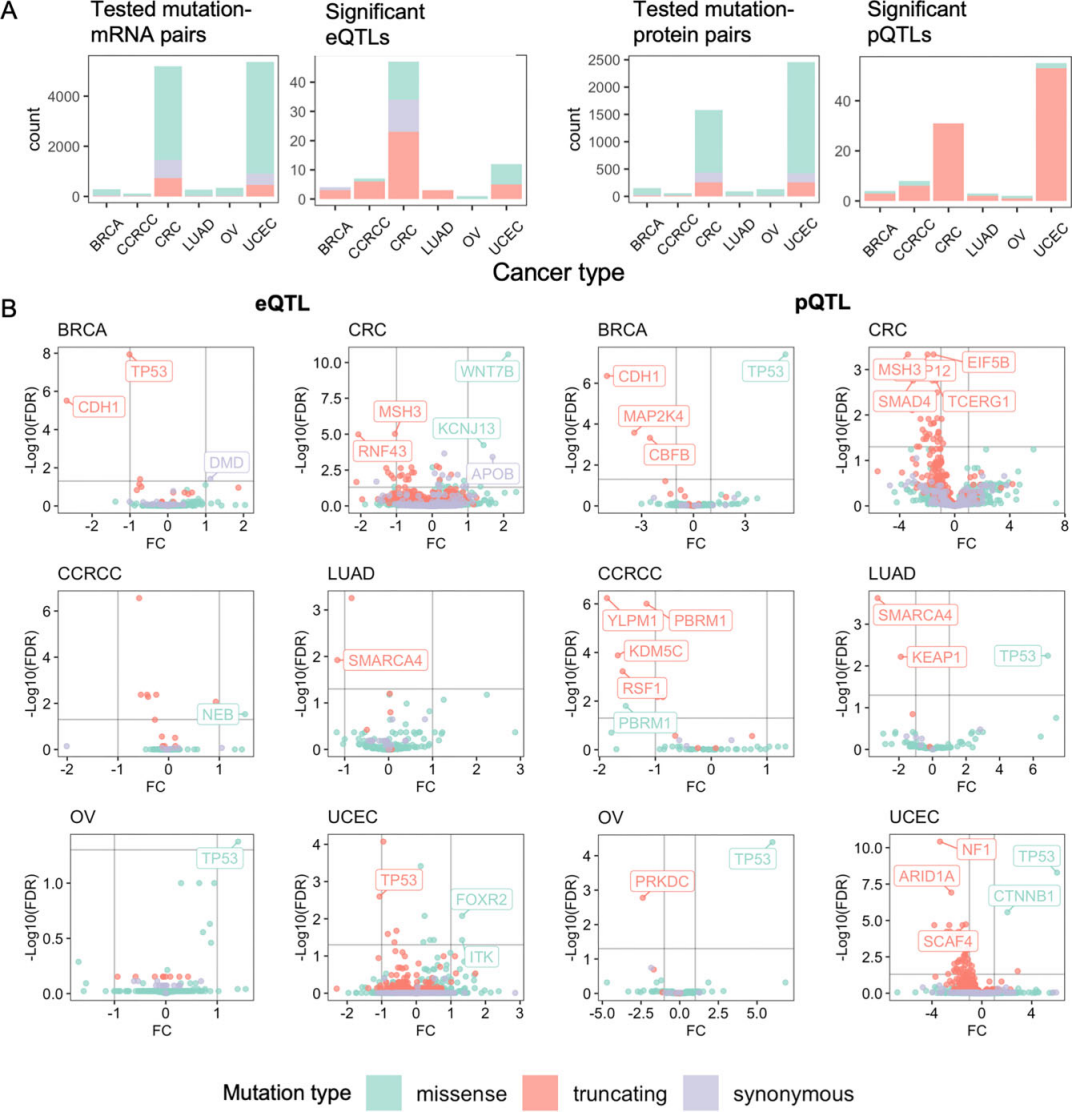
Gene mutations identified as *cis* seQTLs and spQTLs across 6 adult cancer types. (A) Overview of the somatic mutation QTLs identified in different cancer types and mutation types, including missense (green), truncating (orange), and synonymous (purple) mutations. For both eQTLs and pQTLs, the panel on the left shows the counts of the mutation–gene pairs included in analyses, and the figure on the right shows the counts of the significant eQTLs and pQTLs. (B) Volcano plots showing seQTL associations in the 6 cancer types (left) and volcano plots showing spQTL associations (right), where each dot denotes a gene–cancer pair included in the analysis. Top associated genes were further labeled. FC: mRNA/protein expression log fold change; FDR: false discovery rate.

Based on the statistical power achieved by these cohort sizes and to reduce false positives, we focused on genes with 3 or more samples affected by mutations in each functional class of missense, truncation, and synonymous within the cancer cohort, including 134, 13, and 15 genes tested in BRCA; 1,360, 318, and 226 genes tested in CRC; 55, 12, and 4 genes tested in CCRCC; 94, 4, and 8 genes tested in LUAD; 134, 5, and 8 genes tested in OV; and 2,243, 273, and 196 genes tested in UCEC. We sought to identify their seQTLs affecting *cis-*expression (i.e., expression of the mutation-affected genes). Using the multiple regression model (see Methods), we identified 74 gene–cancer seQTL pairs (false discovery rate [FDR] < 0.05), including 4 in BRCA, 47 in CRC, 7 in CCRCC, 3 in LUAD, 1 in OV, and 12 in UCEC (Fig. 2A, Supplementary Table S1). Separated by the functional classes of mutations, 22 of those seQTLs are missense mutations, 12 are synonymous, and 40 are truncating. Top seQTLs showing upregulation of gene expression are primarily missenses, including *SMARCA4* in LUAD, *WNT7B* in CRC, *TP53* in OV, and *FOXR2* in UCEC. Top candidates showing downregulation of gene expression include *TP53* and *CDH1* truncations in BRCA, as well as *TP53* truncations in OV (Fig. 2B).

Using a similar multiple regression but modeling protein abundance as the dependent variable, we identified 103 significant gene–cancer spQTL pairs (FDR < 0.05), including 4 in BRCA, 31 in CRC, 8 in CCRCC, 3 in LUAD, 2 in OV, and 55 in UCEC (Fig. 2A, Supplementary Table S2). Compared to the proportion of gene mutation type evaluated in each cancer type, spQTLs showed significant enrichment for truncations (Fisher exact test *P* < 0.05; Fig. 2A), highlighting the persistent and more profound effect of truncations on protein abundance compared to mRNA levels. Among the identified spQTLs across cancer, 7 are missense and 96 are truncating. For example, truncating mutations of *NF1* and *ARID1A* in UCEC and *YLPM1* in CCRCC are each associated with reduced protein level of the corresponding gene (Fig. 2B). Notably, *TP53* missenses in OV, BRCA, LUAD, and UCEC are each significantly associated with increased protein expression in mutation carriers (Fig. 2B).

To verify these discoveries, we applied the same seQTL and spQTL analyses using retrospective CPTAC data (Supplementary Fig. S1A) that included independent cohorts of BRCA [11], CRC [13], and OV [12] primary tumors. While these cohorts afforded smaller sample sizes, 8 seQTLs and 5 spQTLs were detected in both retrospective and prospective sets. The gene–cancer spQTL pairs showing strong validation in both datasets include *TP53* missense mutations and *CDH1* truncations in BRCA and *TP53* truncations in CRC (Supplementary Fig. S1B).

### Mutations showing concordant effects at mRNA and protein levels

**Figure 3:**
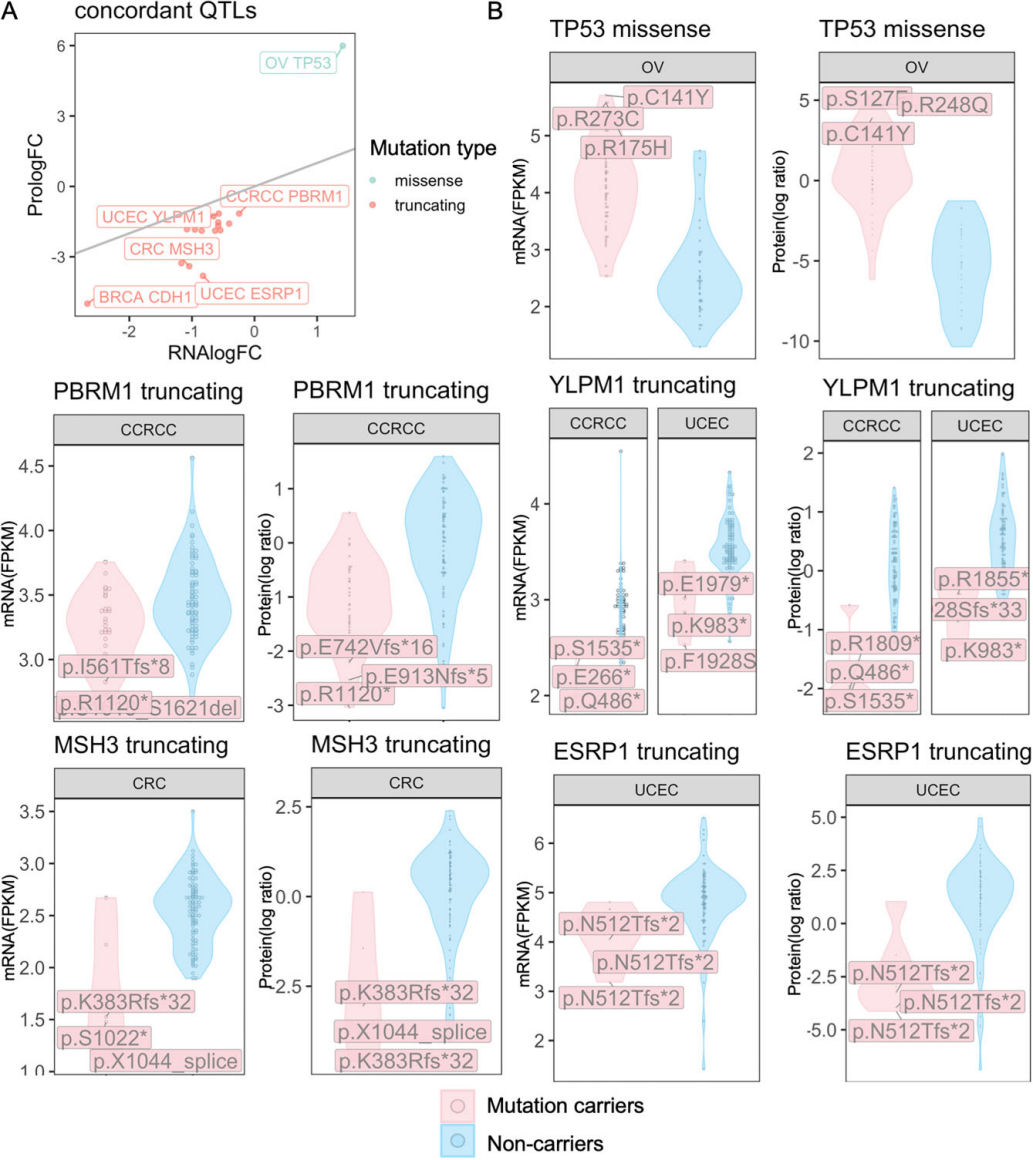
Gene mutations showing concordant impacts on gene and protein expression levels. (A) Overview of concordant QTLs as shown by their effect sizes in log[fold change (FC)], where the gray line shows when the protein logFC equals RNA logFC. Some of the top concordant QTLs were further labeled by cancer type and gene name. (B) Examples of QTL with concordant effects at mRNA and protein expression levels. For each gene, the plot on the left shows the corresponding mRNA levels of mutation carriers versus noncarriers in FPKM, and the plot on the right shows protein level comparison in log ratio (MS TMT measurements) in the respective cancer type labeled on top of each violin plot. The labeled mutations are the 3 mutations whose carriers show the highest absolute expression differences of the mutated gene product compared to the noncarriers.

We next examined the concordance of seQTL and spQTL associations for each gene–cancer type pair. As expected, for most (88.9%) of the significant seQTLs whose genes had sufficient observations at both the mRNA and protein levels, the identified associations showed the same directionality. However, we only identified 17 seQTLs (47.2%) that are also significant spQTLs at an FDR < 0.05, which we show as concordant QTLs (Fig. 3A, Supplementary Table S3). The effect sizes (in log fold change) of these gene–cancer pairs showing concordant seQTLs and spQTLs showed a high correlation between mRNA and protein (Pearson *r* = 0.90, *P* < 7.51E-7).

In different cancer types, genes whose mutation impacts on gene and protein expressions are concordant include well-known drivers of the disease, including *TP53* missense mutations in OV, *CDH1* truncations in BRCA, and *MSH3* truncations in CRC. Upregulation of mutated *TP53* in OV is the only association found for genes affected by missense mutations. The 16 other concordant se/spQTLs are all truncations associated with reduced expression and highlight some “long-tail” driver genes, including *PBRM1* in CCRCC, *YLPM1* in CCRCC/UCEC, and *ESRP1* in UCEC (Fig. 3B). The concordant QTLs with truncating mutation can likely be explained by NMD, which reduces gene expression and in turn diminishes the expression of the corresponding proteins [3] . Compared to the substantially higher counts of seQTL associations (Fig. 2A, B), these concordant se/spQTL effects validate mutation impacts on the gene product.

### Protein-specific mutation impacts not observed at mRNA levels

While most seQTLs and spQTLs show concordance, we postulate that certain mutations may uniquely affect protein abundance but not mRNA levels, which we term somatic protein–specific QTLs (spsQTLs). To identify spsQTLs, we applied 2 methods to stringently retain QTLs with discordant effects at mRNA and protein levels. First, applying a likelihood ratio test (LRT) between 2 regression models of protein level being predicted by mRNA level with or without the mutation term (see Methods) [4], 96 candidate spsQTLs (FDR < 0.05) were identified. Second, complementing this LRT test with an approach filtering for a gene–cancer pair showing significant spQTL (FDR < 0.05) but not seQTLs (see Methods) [23], 86 candidate spsQTLs (FDR < 0.05) were identified.

**Figure 4:**
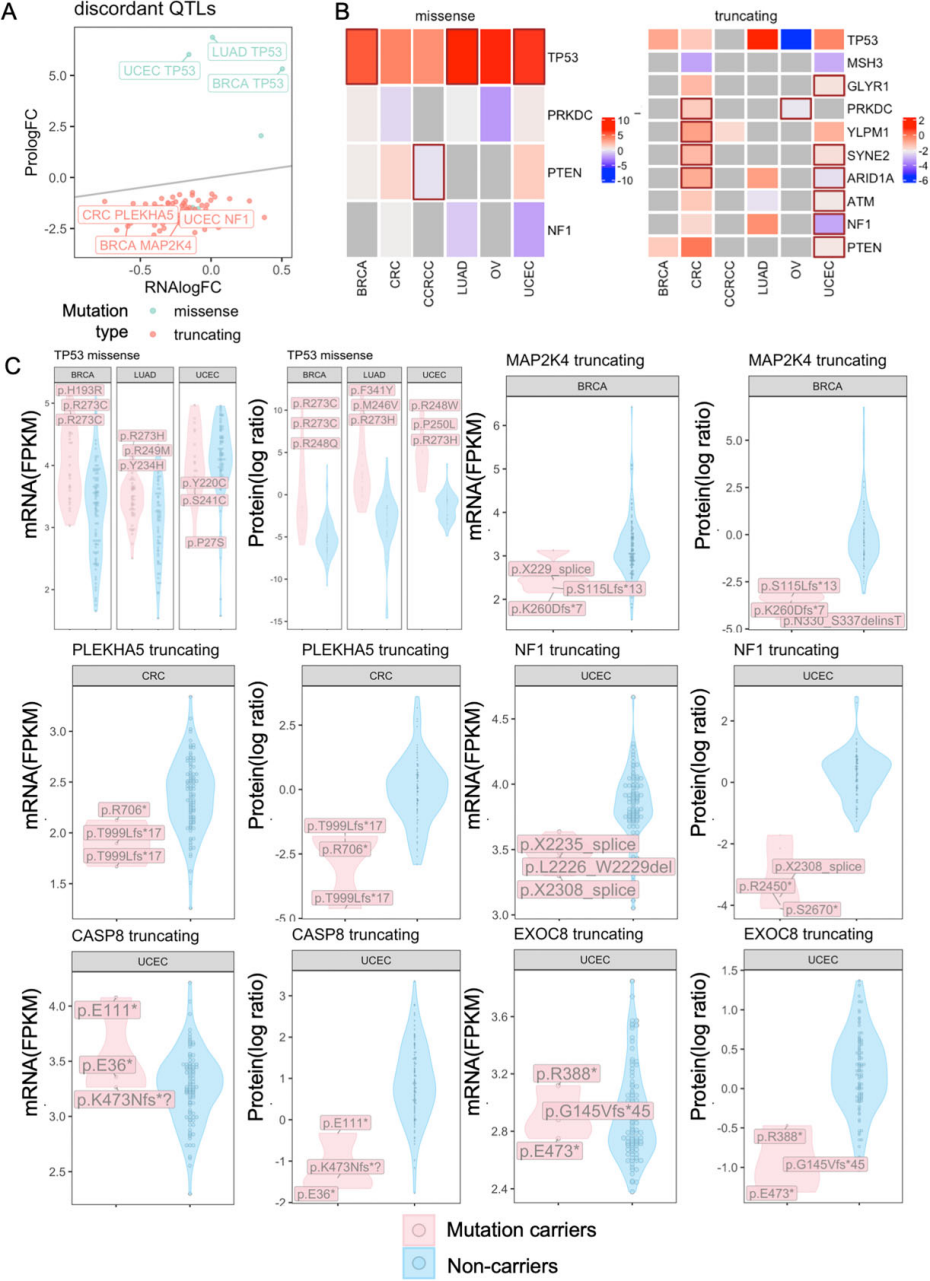
Gene mutations showing discordant impacts on gene and protein expression levels. (A) Overview of discordant QTLs identified by our statistical pipeline, as shown by their effect sizes in logFC, where the gray line shows when the protein logFC equals RNA logFC. (B) Heatmaps of QTLs that are significant as either seQTL or spQTL and that are shared across at least 2 cancer types. Brown box indicates significant spsQTLs, and color indicates the effect size in logFC and average protein expression of mutation carriers in log ratio from the MS TMT quantifications. (C) Examples of QTL with discordant effects at mRNA versus protein levels. For each gene, the plot on the left shows the corresponding mRNA levels of mutation carriers versus noncarriers in FPKM, and the plot on the right shows protein-level comparison in log ratio (MS TMT measurements) in the respective cancer type labeled on top of each violin plot. The labeled mutations are the 3 mutations whose carriers show the highest absolute expression differences of the mutated gene product compared to the noncarriers.

By overlapping candidate spsQTLs identified by both methods, we retained 83 spsQTLs, the majority (92.8%) of which are truncating mutations (Fig. 4A, Supplementary Table S4). Top spsQTLs associated with diminished protein expression include *NF1* truncations in UCEC, *PLEAHK5* truncations in CRC, and *MAP2K4* truncations in BRCA. The only spsQTLs that increase protein expression include *TP53* missense mutations in BRCA, LUAD, and UCEC. (Fig. 4B). We further examined the discordance in mutation impacts on gene and protein expression levels (Fig. 4C). While some of these truncations, such as *NF1* in UCEC and *MAP2K4* in BRCA, were often accompanied by lower-than-median mRNA expression in their respective tumor cohorts, their impacts were strikingly observed at diminished protein expression levels. We highlighted in Supplementary Fig. S2A spsQTLs where the affected gene’s protein showed negative protein log fold-change (logFC), whereas the mRNA logFC is nonnegative, including *CASP8* truncations in UCEC, *ARID1A* truncations in CRC and UCEC, and ATM truncations in LUAD and UCED. We also identified a set of spsQTLs truncations, where the logFC associated with a reduction in proteins is 15 times greater than mRNA’s logFC (Supplementary Fig. S2B). These results suggest that NMD associated with these gene truncations is closely tied to the terminated translation but may not affect mRNA expression to the same degree [24].

To complement the cross-tumor analyses, we also utilized the CPTAC samples with paired tumor-normal tissues to conduct paired differential expression tests for both protein and mRNA expression (Fig. 1A). The paired sample sizes with proteomic data include 17 in BRCA, 17 in UCEC, 84 in CCRCC, 100 in LUAD, 29 in CRC, and 10 in OV (Fig. 1B). Covariates including age at diagnosis, ethnicity, race, and sequencing operator are adjusted in the analysis. While this analysis had varied statistical power due to different normal tissue availabilities across cancer types, it served as an independent validation of spQTLs (Supplementary Table S5). This paired tumor-normal analysis validated the protein-level impacts of several discordant spsQTLs (Supplementary Fig. S3A) as well as some concordant se/spQTLs (Supplementary Fig. S3B). For example, the validated discordant spsQTLs include truncations of *SMAD4* and *SCRIB* in CRC as well as *NF1, GLYR1*, and *RASA1* in UCEC (Supplementary Fig. S3A). The validated concordant se/spQTLs include truncations of *YLPM1* and *PBRM1* in CCRCC, *SMARCA4* and *KEAP1* in LUAD, and *ESRP1* as well as *JAK2* in UCEC (Supplementary Fig. S3B).

### Functional evidence of *TP53* missenses associated with high protein expression

**Figure 5:**
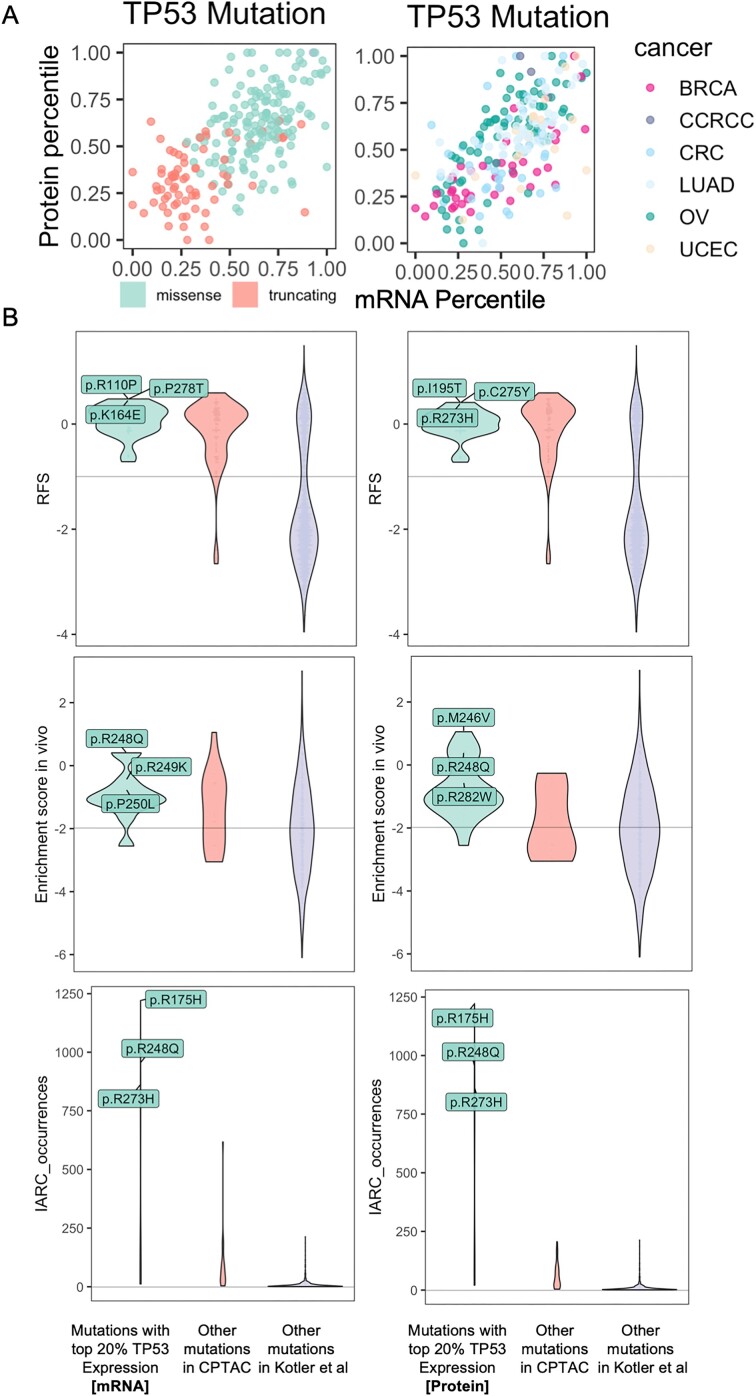
Functional verification of *TP53* mutation associated with high mRNA or protein levels using *in vitro* and *in vivo* data from a MAVE experiment. (A) Percentile of averaged expression associated with a given *TP53* mutation at the mRNA (x-axis) and protein (y-axis) levels in the respective cancer cohort. *TP53* mutations are color-coded by mutation type (left) and observed cancer type (right), respectively. (B) Violin plots comparing the *in vitro* functional score (RFS, top), *in vivo* enrichment score (middle), and IARC occurrences (bottom) for TP53 mutations in the 3 groups defined by (i) *TP53* mutations with top 20% mRNA (left) or protein (right) expression in the prospective CPTAC cohorts, (ii) the other *TP53* mutations observed across all CPTAC samples, and (iii) the rest of the assayed *TP53* mutations from Kotler et al. [21].

Notably, *TP53* missenses are associated with higher protein expression in multiple cancer cohorts, in addition to the expected reduction in expression associated with truncations (Fig. 5A). Such *cis*-effect of functional *TP53* missense mutations had previously been observed through immunohistochemistry (IHC [25]) or MS global proteomics experiments [26]. Here, we hypothesized that functional *TP53* missense mutations are more likely to show high levels of concurrent protein-level expression in the mutated tumor sample. To test this hypothesis, we compared gene- and protein-level *TP53* expression from CPTAC with *TP53* mutation-level functional data from the *in vitro* and *in vivo* MAVE experiment conducted by Kotler et al. [21], who designed a p53 variants library to study the functional impact of those mutations.

We divided the *TP53* missense mutations from Kotler et al. [21] into 3 categories: (i) *TP53* mutations with top 20% mRNA or protein expression in the prospective CPTAC cohorts, (ii) the other *TP53* mutations observed across all CPTAC samples, and (iii) the rest of the assayed *TP53* mutations from Kotler et al. [21]. For *in vitro* data, the number of tested mutations by each category is 32, 78, and 1,033, respectively. For *in vivo* data, the number of tested mutations by each category is 19, 10, and 381, respectively. We first compared the relative fitness score (RFS) measured from the *in vitro* assays [21] . While there may be a trend, we did not observe a significant difference between all the other mutations versus *TP53* missenses associated with either top 20% expression based on either mRNA (*P* = 0.090, Wilcoxon rank-sum test) or protein expression (*P* = 0.720).

We next compared the *in vivo* enrichment scores across the same categories and found that *TP53* missenses associated with top 20% protein expression showed a significantly higher enrichment score *in vivo* compared to that of other *TP53* missenses found in CPTAC (*P* = 0.016) or other experimentally measured *TP53* mutations (*P* = 3.23E-5, Fig. 5B, Supplementary Table S6). In comparison, *TP53* missenses associated with top 20% mRNA expression did not show a significant *in vivo* score difference to that of other *TP53* missenses found in CPTAC (*P* = 0.170). Kotler et al. [21] observed that there was no significant correlation between enrichment score *in vivo* and RFS *in vitro*, which is consistent with our observations and may be explained by the different selective pressures between these settings *in vivo* and *in vitro*. Finally, *TP53* missenses associated with top 20% protein expression (*P* = 5.91E-7) or top 20% mRNA expression (*P* = 2.38E-2) showed significantly higher prevalence than other CPTAC mutations based on counts from the International Agency for Research on Cancer (IARC) database [21] (Fig. 5B, Supplementary Table S6). Overall, these analyses suggested that protein-level consequences from primary tumor samples can aid the identification of functional mutations.

## Discussion

Herein, we analyzed how somatic mutations affect mRNA and protein levels using matched genomic, transcriptomic, and global proteomic data from 953 cases across 6 solid cancer types. We first investigated the mutation impacts at the mRNA level and protein level, finding that although most seQTLs have the same direction of effect as spQTLs, less than half of them are also significant at the protein level. We also studied the concordant or discordant relationship between seQTL versus spQTLs, finding several spsQTLs that have disproportional effects on protein. Finally, we conducted analyses to provide functional validation [21] for our findings of TP53 missenses associated with high protein expression.

Integrating protein-level data identified nearly 47.2% seQTLs as concordant, significant spQTLs. The result demonstrates the capacity of proteomic data to validate genomic findings and potentially filter out noises that may arise, for example, due to the more transient nature of transcription compared to translation. In addition to well-known tumor suppressors like *TP53* and *MSH3*, other gene mutations with concordant effects may also be “long-tail” driver genes that will otherwise require large cohort sample sizes to discover. For example, *PBRM1*, which we found in CCRCC, is a subunit of the PBAF chromatin remodeling complex thought to be a tumor suppressor gene whose mutations may confer synthetic lethality to DNA repair inhibitors [27]. *ESRP1*, found in UCEC, is crucial in regulating alternative splicing and the translation of some genes during organogenesis [28]. Other less-studied genes we identified include *YLPM1* truncations associated with concordantly reduced *YLPM1* mRNA and protein expression levels in both CCRCC and UCEC. Analyzing the distribution of these gene mutations on NCI’s Genome Data Commons, we observed many other recurrent truncations (Supplementary Fig. S4), suggesting these mutations may represent some of the “long-tail” driver mutations that warrant further investigation [29, 30].

By devising a specific pipeline to detect spsQTLs, our results showed that apart from mutations that influence protein level mediated by changes in mRNA level, many mutations are associated with disproportional aberrations at the protein level compared to mRNA changes, indicating posttranscriptional regulation. SpsQTLs were found to affect known driver genes such as *TP53* missenses and truncations in *NF1* [31] and *MAP2K4* [32]. In most cases, protein molecules are more direct mediators of cellular functions and phenotypes than mRNAs [33]. Thus, the discordant effect between mRNA level and protein level discovered in our study highlights the importance of exploring disease mechanisms and developing treatments at the protein level.

One possible source of spsQTLs is the imperfect correlation between mRNA and protein expression in the affected genes. Additional statistical analyses revealed that these mRNA–protein correlations range widely across genes and cancer types (Supplementary Fig. S5). While genes harboring spsQTLs have lower mRNA–protein correlations in general than genes with concordant eQTL and pQTL, this is not the case for several discordant genes, including *MAP2K4* in BRCA and *PBRM1* in CCRCC (Supplementary Table S7). Based on the number of mutations and genes identified, CRC and UCEC reached statistically significant differences between concordant and all other expressed genes (Wilcoxon rank-sum tests, *P* = 0.0056 and *P* = 0.022, respectively); in CRC, mRNA–protein correlations also showed significant differences between discordant and all other expressed genes (*P* = 0.013 and *P* = 0.29, respectively); other cancer types likely did not reach statistical significance likely due to sufficient mutations identified. The imperfect correspondence between gene mRNA–protein correlations and mutation impacts further stresses the need to analyze and consider protein-specific impacts of mutations. Supplementary Table S7 provides complete mRNA–protein correlation data for all concordant/discordant eQTL/pQTLs in their respective cancer type for in-depth examination.

This study has several limitations. First, our findings do not distinguish between several potential mechanisms that could lead to discordant effects of mutations on gene and protein expression. One possibility is that the mutation affects the efficiency of translation, leading to changes in protein levels that are not reflected in mRNA levels. For example, accumulating evidence in recent years suggests that NMD is closely tied to the termination of translation [24], which may explain instances when some truncations afford much stronger associations with protein levels in our findings. But, in many cases, the mechanisms of how mutations may affect protein abundance may be context and gene specific and remain to be elucidated. For example, certain mutations may influence the binding of RNA-binding proteins and the efficiency of translation, whereas others may alter posttranslational modifications, such as phosphorylation or ubiquitination, which can impact protein stability or degradation without affecting transcription or translation rates. Second, the proteogenomic tumor cohorts used herein, while being some of the largest studies to date, still are limited in sample sizes and preclude sufficient statistical power to identify pQTLs at a single mutation level or reveal *trans* effects. Third, given the limitation of current omic technology and data, our findings do not resolve mutation impact on proteins at the temporal, spatial, or single-cell resolution but provide candidate mutations to be investigated in future studies. Fourth, our regression models assume a linear relationship between mutations (1 gene at a time), confounders, and expression, which may not capture more complex, nonlinear effects of mutations on multiple mRNA or protein expression. Future studies could explore nonlinear regression models or neural network approaches to better account for these effects. Fifth, we employed 2 complementary methods to confidently identify spsQTLs that represent true protein-specific regulatory events. However, the reliance on FDR thresholds could still limit the detection of spsQTLs with subtle effects. Alternative approaches, such as Bayesian models that account for prior biological knowledge or hierarchical modeling, could be considered in future analyses to improve the specificity of spsQTL detection. Additionally, while our method focuses on *cis*-acting mutations, potential *trans*-acting effects could be missed, a limitation that should be explored in larger datasets or by incorporating network-based analyses.

Finally, using *TP53* missense mutations as an example, we showed that protein-level expression can serve as an effective strategy to prioritize functional mutations. As DNA-seq becomes ever more commonplace, many rare mutations are being identified, and it remains challenging to accurately classify their functional impacts. Our data demonstrated that *TP53* missenses associated with high protein expression show significantly higher functional scores, particularly those measured *in vivo*. This protein expression–based prioritization strategy can be particularly powerful when combined with high-throughput functional assays like using MAVE model systems that are typically *in vitro*. Considering that both MAVE and proteogenomic datasets of tumor cohorts are both expanding quickly in the next few years [34, 35], the combined approaches can help effectively pinpoint functional mutations for mechanistic and clinical characterization. The prioritized mutations based on protein-level consequences may also guide the selection of targeted therapy to advance precision medicine.

## Methods

### Proteogenomic datasets

The prospective CPTAC data were downloaded and processed as described in the Method section of the work of Elmas et al. [36]. The overview table in Fig. 1A of the dataset describes, for each cancer cohort, the sample size, female patient percentage, average cancer onset age, and tumor stage. Samples are normalized by their median absolute deviations (MAD), so that the MAD of all samples in the dataset is 1. Protein markers with high fractions (greater than 20%) of missing values are filtered out. For the corresponding RNA-seq data, we used the log2 normalization on the FPKM (fragments per kilobase of exon per million mapped fragments)–normalized RNA-seq counts, and genes that have no expression in at least 90% of the samples were filtered out.

The proteomics data used for validation were downloaded from the NCI CPTAC portal [37]. The dataset overview table in Supplementary Fig. S1A describes for each cancer cohort the sample size, female patient percentage, average cancer onset age, and tumor stage. The validation data are processed in the same way as the prospective data. The RNA-seq datasets of the 3 retrospective CPTAC cohorts were downloaded from the NCI CPTAC DCC portal [37]. The RNA expression was measured in FPKM)[38, 39] and was further normalized by log2(FPKM + 1).

### pQTL and eQTL identification

For each cancer cohort, we identified pQTLs and eQTLs using the multiple linear regression model as implemented in the “limma” R package (v3.42.2) [40]. We also corrected confounding factors, including age, gender, ethnicity, and TMT batch. The FDR was corrected from the *P* values with the Benjamini–Hochberg procedure [41], ensuring that the identified QTLs are statistically robust. Somatic mutations are grouped at a gene level in the multiple regression model, similar to that implemented by our previously developed AeQTL tool [7]. Mutations are separately analyzed by their mechanisms of action, including nonsynonymous mutations that likely do not affect expression, missense mutations, and truncating mutations—including frameshift and in-frame indels, nonsense, splice site, and translation start site mutations. To improve statistical power, we focused our analysis on genes with 3 or more mutations in each cancer cohort and analyzed associations of mutations affecting *cis-*expression of the corresponding mRNA or protein products.

### spsQTL identification

We combined 2 complementary statistical methods to identify spsQTLs. In the first method adopted from Battle et al. [4], we compared the following 2 nested linear models using the LRT with the “anova” function in R:


\begin{eqnarray*}
p\; = \;\mu \; + \;{\beta _0}g\; + \;\;{\beta _1}r
\end{eqnarray*}

\begin{eqnarray*}
p\; = \;\mu \; + \;\;{\beta _2}r
\end{eqnarray*}

where $g\;$ is the genotype, $r$ represents RNA level, and p is the protein level. By comparing these models using the LRT and filtering results with an FDR less than 0.05, we identified candidate spsQTLs where the genotype (mutation) has a disproportionate impact on protein abundance independent of mRNA expression.

In the second method adopted from Mirauta et al. [23], we selected QTLs where the spQTL FDR was less than 0.05 but the corresponding seQTL FDR was greater than 0.05 as candidate spsQTLs to specifically identify mutations that affect protein levels without influencing mRNA. We then overlapped these 2 lists of candidate spsQTLs obtained from 2 complementary methods to identify the final list of spsQTLs for downstream analyses.

### mRNA–protein correlation

To investigate the impact of mutations on mRNA and protein expression, we performed a comparative analysis across the 6 solid cancer types. For each cancer type, Pearson correlation coefficients were calculated for individual genes using paired mRNA and protein expression data. We analyzed 3 groups of genes we identified as showing a variable impact on mRNA/protein-level expressions: concordant genes (with mutations showing concordant effects at both mRNA and protein levels in *cis*), discordant genes (showing protein-specific effects), and other genes (showing no concordant or protein-specific impact). Our aim was to test the hypothesis whether the mRNA–protein correlations of the concordant/discordant groups differed from the baseline genome-wide mRNA–protein correlations, indicating biological significance. To assess this, we employed a 2-sample Wilcoxon rank-sum test, comparing the mRNA–protein correlations for the concordant/discordant and other gene groups within each cancer type. Pairwise comparisons were made between the concordant and other gene sets, as well as between the discordant and other gene sets, demonstrating that the correlation coefficients for these groups were drawn from distinct population distributions with statistical significance at a *P* value threshold of 0.05.

### Tumor-normal differential expression analysis

We conducted this analysis in the prospective CPTAC cohorts with paired tumor-adjacent tissue-normal samples. For each cancer cohort, we paired the tumor and normal samples from the same patient and performed a differential protein/mRNA expression analysis to identify differentially expressed proteins with the “limma” package. Demographic factors and batch effects, including age, ethnicity, race, and sequencing operator, are adjusted in the multiple regression model.

## Availability of Supporting Source Code and Requirements

Project name: Protein expression quantitative trait loci (pQTLs): software and analytic code

Project homepage: https://github.com/Huang-lab/pQTL [42]

Operating system(s): Platform independent

Programming language: R, Python, Jupyter Notebook

License: MIT

## Additional Files

**Supplementary Fig. S1**. Overview of the retrospective cohorts. (A) Summary of the retrospective CPTAC proteogenomic cohorts used for the discovery analyses, including cancer type abbreviation, data source, sample size of tumor (T) and normal (N) tissues, female percentage, average onset age in years, and tumor stage distribution. (B) Volcano plots showing seQTL associations in the 6 cancer types (left) and volcano plots showing spQTL associations (right), where each dot denotes a gene–cancer pair included in the analysis. Top associated genes were further labeled. FC: log fold change; FDR: false discovery rate.

**Supplementary Fig. S2**. spsQTLs with strong effects. (A) Examples of spsQTL whose effect sizes in mRNA level and protein level are in a different direction. For each gene, the plot on the left shows the corresponding mRNA levels of mutation carriers versus noncarriers in FPKM, and the plot on the right shows protein-level comparison in log ratio (MS TMT measurements) in the respective cancer type labeled on top of each of the violin plots. The labeled mutations are the 3 mutations whose carriers show the highest absolute expression differences of the mutated gene product compared to the noncarriers. (B) Examples of spsQTL with a protein logFC and mRNA logFC ratio greater than 15.

**Supplementary Fig. S3**. Overlap of significant QTLs in cross-tumor analysis and matched tumor-normal analysis projected onto pQTL volcano plots based on cross-tumor analyses. The plots were made separately for (A) discordant spsQTLs and (B) concordant eQTL/pQTLs.

**Supplementary Fig. S4**. Example lolliplots showing mutations for 2 genes that were identified as spsQTLs, including YLPM1 and ESRP1. The number on each disc denotes the number of mutations in that position, and the color of the disc represents the mutation type.

**Supplementary Fig. S5**. Correlation coefficients of concordant versus discordant genes. The violin plots depict the distribution of correlation coefficients between matched mRNA and protein expressions for concordant (blue), discordant (red), and other genes (gray) across the 6 cancer types studied. Genes with notable correlations are labeled in each plot.

**Supplementary Table S1**. List of expression quantitative trait loci (eQTLs) identified across 6 cancer types. This table provides details on the gene mutations associated with mRNA expression levels, including statistical test results, mutation type, *P* values (adjusted), and effect sizes.

**Supplementary Table S2**. List of protein quantitative trait loci (pQTLs) identified across 6 cancer types. This table provides details on the gene mutations associated with protein abundance levels, including statistical test results, mutation type, *P* values (adjusted), and effect sizes.

**Supplementary Table S3**. Concordant expression and protein quantitative trait loci (eQTLs and pQTLs) identified across 6 cancer types. This table includes information on the gene mutations, identified cancer types, and their impact on both mRNA and protein expression levels, demonstrating loci with consistent effects across both molecular layers.

**Supplementary Table S4**. Significant somatic protein-specific QTLs (spsQTLs) identified by our statistical pipeline across 6 cancer types. This table details the loci with mutations showing significant impacts on protein abundance not explained by mRNA levels, including summary statistics for eQTL/pQTL tests and the LRT and overlap test results.

**Supplementary Table S5**. Summary statistics for differentially expressed proteins (DEPs) identified in paired tumor-normal (TN) samples across 6 cancer types. This table includes the test statistics of protein expression differences between tumor and normal tissues harboring the specific mutation.

**Supplementary Table S6**. Test statistics between the 3 groups of TP53 mutations. The tested groups were defined by (i) TP53 mutations with top 20% mRNA (left) or protein (right) expression in the prospective CPTAC cohorts, (ii) the other TP53 mutations observed across all CPTAC samples, and (iii) the rest of the assayed TP53 mutations from Kotler et al. using *TP53* functional scores form Kotler et al.

**Supplementary Table S7**. Pearson’s correlation coefficient tests between paired mRNA and protein expressions for each concordant and discordant gene, within each cancer cohort.

giae113_Supplemental_Files

giae113_GIGA-D-24-00168_Original_Submission

giae113_GIGA-D-24-00168_Revision_1

giae113_GIGA-D-24-00168_Revision_2

giae113_Response_to_Reviewer_Comments_Original_Submission

giae113_Response_to_Reviewer_Comments_Revision_1

giae113_Reviewer_1_Report_Original_Submission Jason Li --7/25/2024

giae113_Reviewer_2_Report_Original_Submission Yuan Liu --7/31/2024

giae113_Reviewer_2_Report_Revision_1 Yuan Liu --9/22/2024

## Data Availability

Proteomic data for CPTAC-2/3 cohorts can be found on National Cancer Institute (NCI) Proteomic Data Commons (PDC) [43]. The studies used in the discovery cohorts and their PDC study IDs are BRCA (PDC000120), CRC (PDC000116), CCRCC (PDC000127), LUAD (PDC000153), OV (JHU: PDC000110; PNNL: PDC000118), and UCEC (PDC000125). The studies used in the validation cohorts and their PDC study IDs are BRCA (PDC000173), CRC (PDC000111), and OV (JHU: PDC000113; PNNL: PDC000114). Genomic data, including DNA mutation and transcriptome profiling for all CPTAC-2/3 cohorts used herein, can be found on National Cancer Institute (NCI) Genome Data Commons (GDC) [44] (dbGaP Study Accession #: phs000892) and (dbGaP Study Accession #: phs001287) [45]. Data for TP53 MAVE assays can be downloaded from the Supplementary Information from Kotler et al. [21]. Supporting data of our analysis results and an archival copy of the corresponding code are available via the *GigaScience* repository, GigaDB [46].
