# Supplementary material for: Mutation impact on mRNA versus protein expression across human cancers
Source: Gigascience. 2025 Jan 6;14:giae113. doi: 10.1093/gigascience/giae113 (PMC11702362; doi:10.1093/gigascience/giae113)
Supplement: giae113_Supplemental_Files [file giae113_supplemental_files.zip › SuppFigures.pdf]

A

Cancer Type	Breast Cancer	Colorectal Cancer	Ovarian Cancer
Abbreviation	**BRCA**	**CRC**	**OV**
Data Source	Mertins et al. 2016 (PMID: 27251275)	Zhang B et al. 2014 (PMID: 25043054)	Zhang H et al. 2016 (PMID: 27372738)
Sample Size (Tumors/Normals)	T: 77	T: 90	T: 173<br>N: 1
Female %	97.4%	46.7%	100%
Average Onset (yr)	58.9	69.4	60.8
Tumor Stage	1: 9.1%<br>2: 64.9%<br>3: 23.4%<br>4: 1.3%	1: 16.7%<br>2: 42.2%<br>3: 28.9%<br>4: 12.2%	1: 1.7%<br>2: 5.7%<br>3: 76.3%<br>4: 16.1%

B

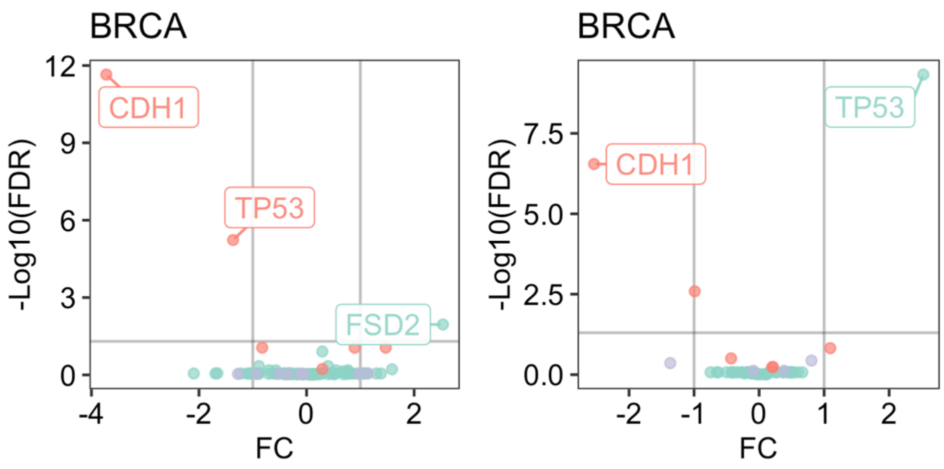

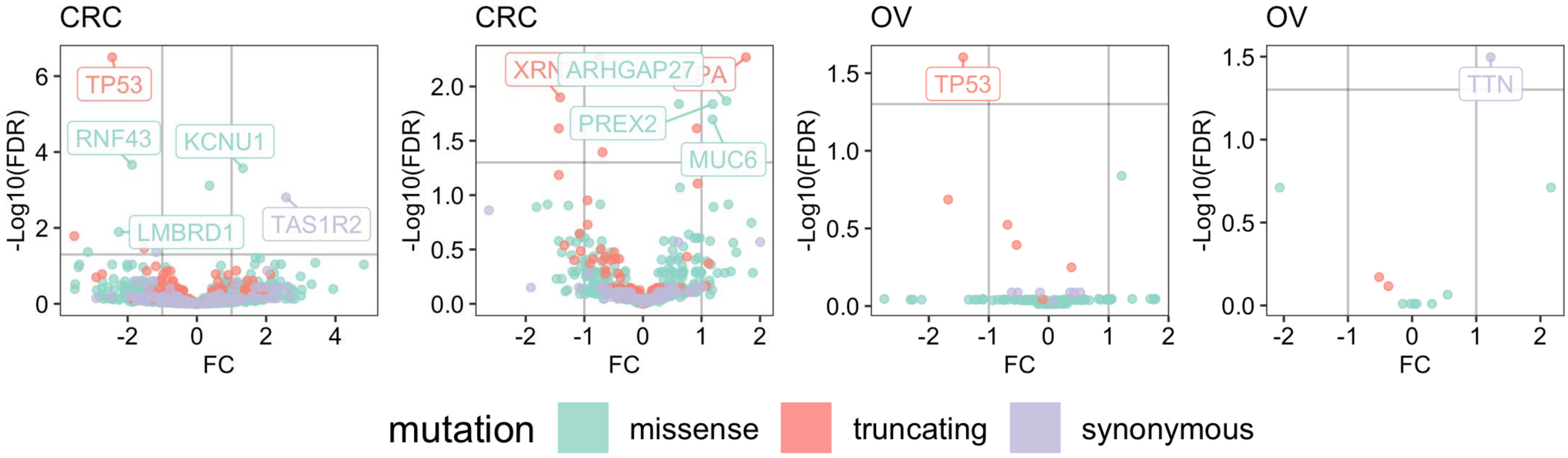

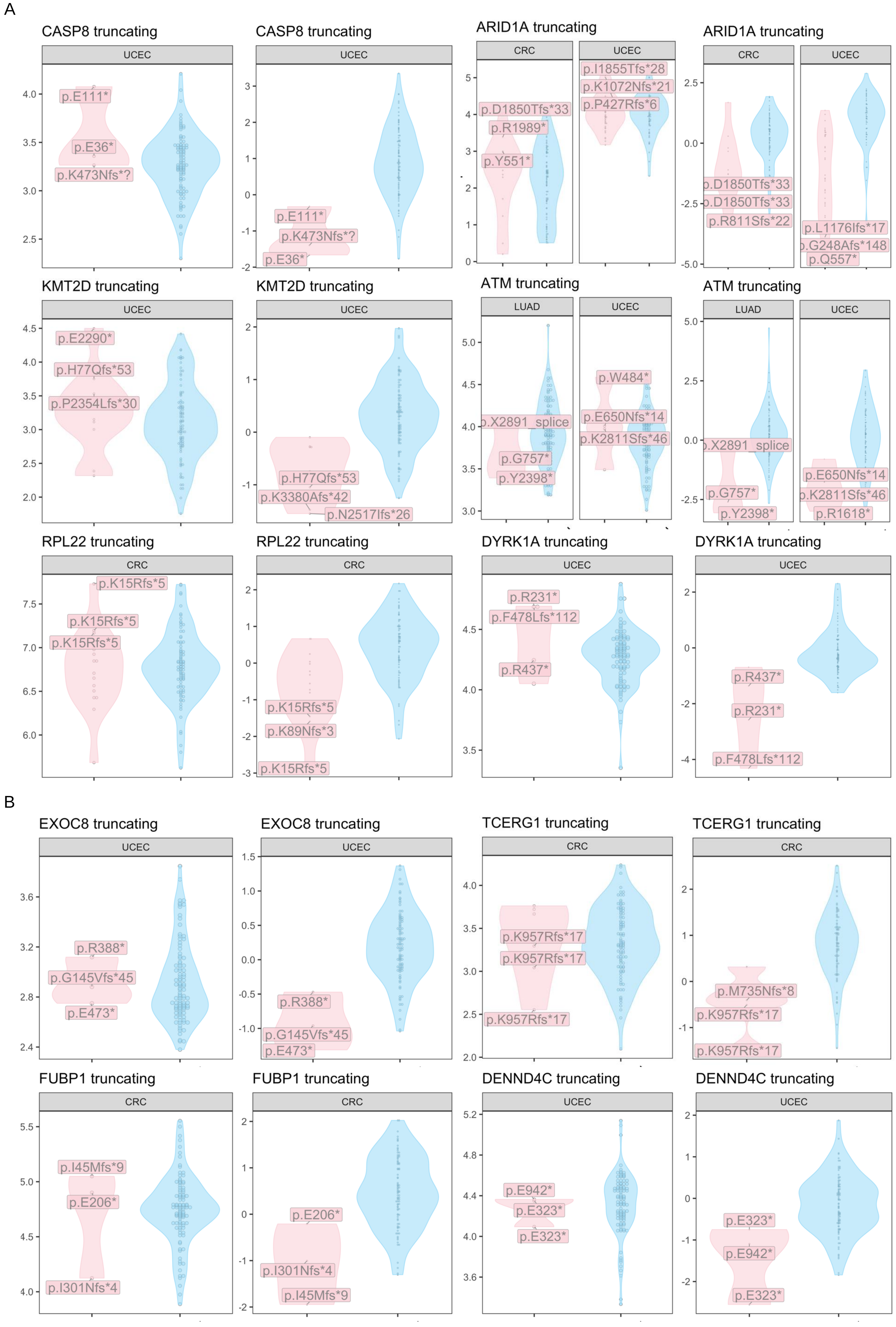
A
CASP8 truncating
UCEC
p.E111*
p.E36*
p.K473Nfs*?
CASP8 truncating
UCEC
p.E111*
p.K473Nfs*?
p.E36*
ARID1A truncating
CRC
UCEC
p.D1850Tfs*33
p.R1989*
p.Y551*
p.I1855Tfs*28
p.K1072Nfs*21
p.P427Rfs*6
ARID1A truncating
CRC
UCEC
p.D1850Tfs*33
p.D1850Tfs*33
p.R811Sfs*22
p.L1176Ifs*17
p.G248Afs*148
p.Q557*
KMT2D truncating
UCEC
p.E2290*
p.H77Qfs*53
p.P2354Lfs*30
KMT2D truncating
UCEC
p.H77Qfs*53
p.K3380Afs*42
p.N2517Ifs*26
ATM truncating
LUAD
UCEC
p.X2891_splice
p.G757*
p.Y2398*
p.W484*
p.E650Nfs*14
p.K2811Sfs*46
ATM truncating
LUAD
UCEC
p.X2891_splice
p.G757*
p.Y2398*
p.E650Nfs*14
p.K2811Sfs*46
p.R1618*
RPL22 truncating
CRC
p.K15Rfs*5
p.K15Rfs*5
p.K15Rfs*5
RPL22 truncating
CRC
p.K15Rfs*5
p.K89Nfs*3
p.K15Rfs*5
DYRK1A truncating
UCEC
p.R231*
p.F478Lfs*112
p.R437*
DYRK1A truncating
UCEC
p.R437*
p.R231*
p.F478Lfs*112
B
EXOC8 truncating
UCEC
p.R388*
p.G145Vfs*45
p.E473*
EXOC8 truncating
UCEC
p.R388*
p.G145Vfs*45
p.E473*
TCERG1 truncating
CRC
p.K957Rfs*17
p.K957Rfs*17
p.K957Rfs*17
TCERG1 truncating
CRC
p.M735Nfs*8
p.K957Rfs*17
p.K957Rfs*17
FUBP1 truncating
CRC
p.I45Mfs*9
p.E206*
p.I301Nfs*4
FUBP1 truncating
CRC
p.E206*
p.I301Nfs*4
p.I45Mfs*9
DENND4C truncating
UCEC
p.E942*
p.E323*
p.E323*
DENND4C truncating
UCEC
p.E323*
p.E942*
p.E323*

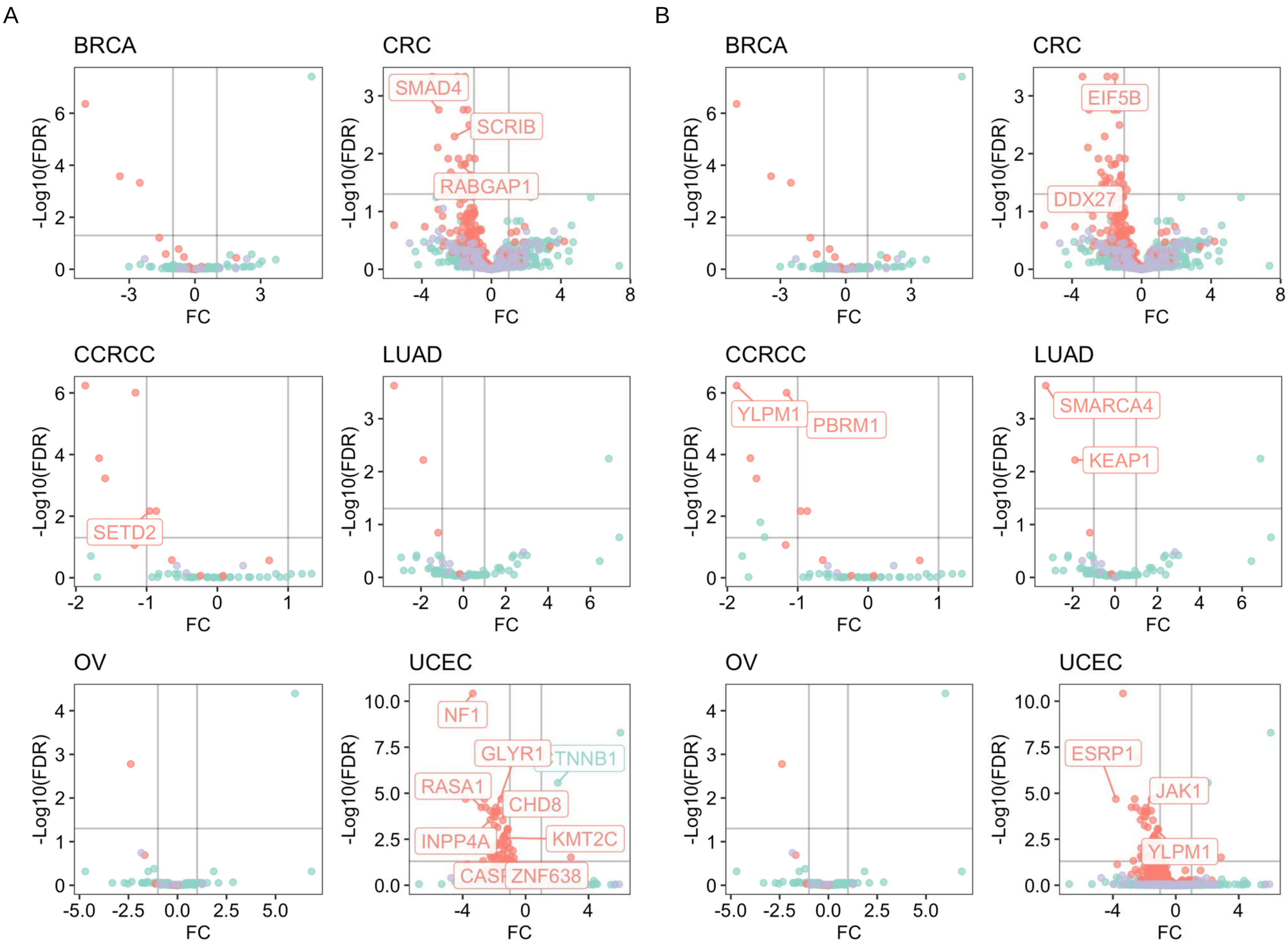
A
B
BRCA
CRC
CCRCC
LUAD
OV
UCEC
-Log10(FDR)
FC
SMAD4
SCRIB
RABGAP1
SETD2
NF1
GLYR1
CTNNB1
RASA1
CHD8
INPP4A
KMT2C
ZNF638
EIF5B
DDX27
YLPM1
PBRM1
SMARCA4
KEAP1
ESRP1
JAK1

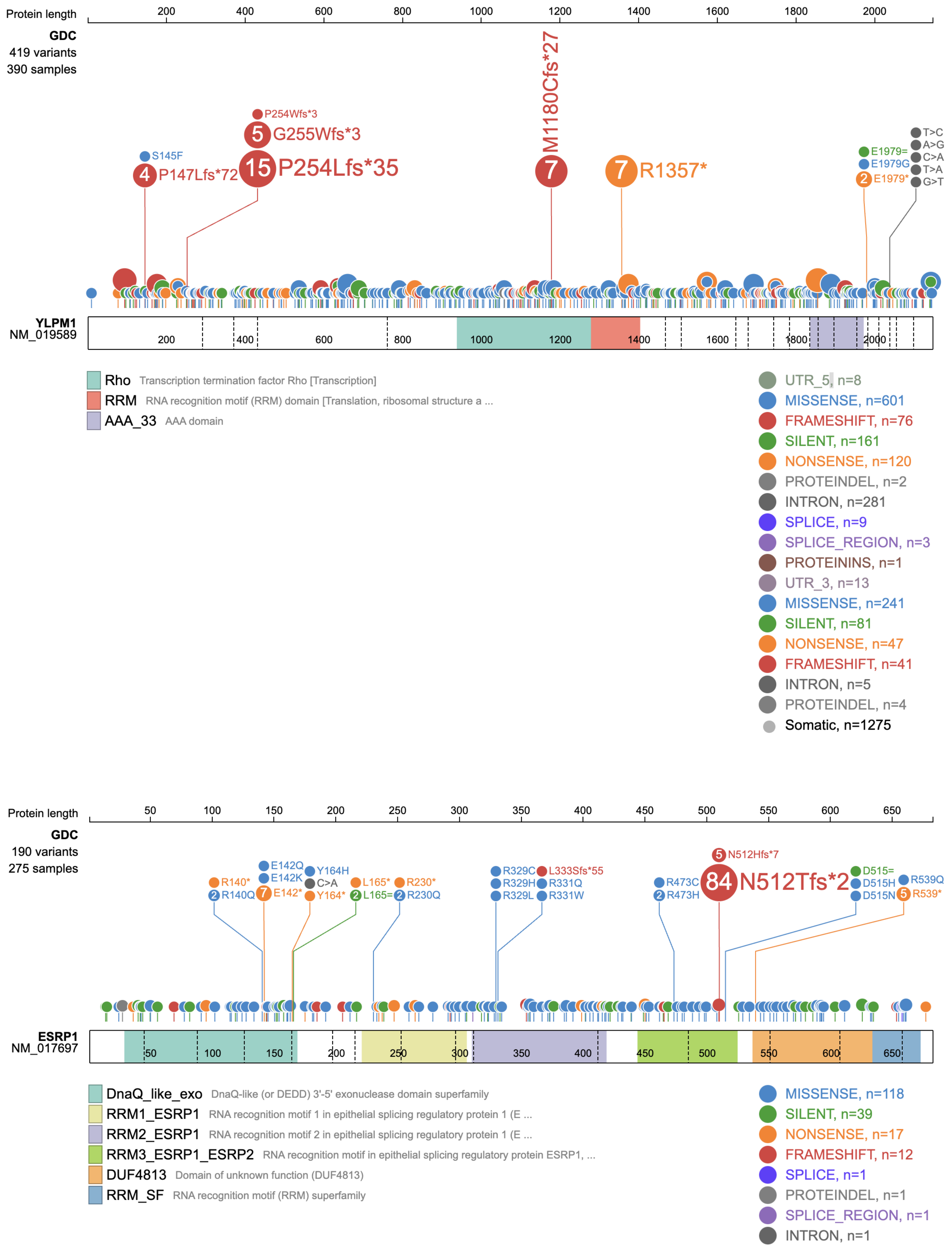

Protein length
GDC
419 variants
390 samples
P254Wfs*3
5 G255Wfs*3
S145F
4 P147Lfs*72
15 P254Lfs*35
M1180Cfs*27
7
7 R1357*
E1979=
E1979G
2 E1979*
T>C
A>G
C>A
T>A
G>T
YLPM1
NM_019589
Rho Transcription termination factor Rho [Transcription]
RRM RNA recognition motif (RRM) domain [Translation, ribosomal structure a ...
AAA_33 AAA domain
UTR_5, n=8
MISSENSE, n=601
FRAMESHIFT, n=76
SILENT, n=161
NONSENSE, n=120
PROTEINDEL, n=2
INTRON, n=281
SPLICE, n=9
SPLICE_REGION, n=3
PROTEININS, n=1
UTR_3, n=13
MISSENSE, n=241
SILENT, n=81
NONSENSE, n=47
FRAMESHIFT, n=41
INTRON, n=5
PROTEINDEL, n=4
Somatic, n=1275
Protein length
GDC
190 variants
275 samples
R140*
2 R140Q
E142Q
E142K
7 E142*
Y164H
C>A
Y164*
L165*
2 L165=
R230*
2 R230Q
R329C
R329H
R329L
L333Sfs*55
R331Q
R331W
R473C
2 R473H
5 N512Hfs*7
84 N512Tfs*2
D515=
D515H
D515N
R539Q
5 R539*
ESRP1
NM_017697
DnaQ_like_exo DnaQ-like (or DEDD) 3'-5' exonuclease domain superfamily
RRM1_ESRP1 RNA recognition motif 1 in epithelial splicing regulatory protein 1 (E ...
RRM2_ESRP1 RNA recognition motif 2 in epithelial splicing regulatory protein 1 (E ...
RRM3_ESRP1_ESRP2 RNA recognition motif in epithelial splicing regulatory protein ESRP1, ...
DUF4813 Domain of unknown function (DUF4813)
RRM_SF RNA recognition motif (RRM) superfamily
MISSENSE, n=118
SILENT, n=39
NONSENSE, n=17
FRAMESHIFT, n=12
SPLICE, n=1
PROTEINDEL, n=1
SPLICE_REGION, n=1
INTRON, n=1

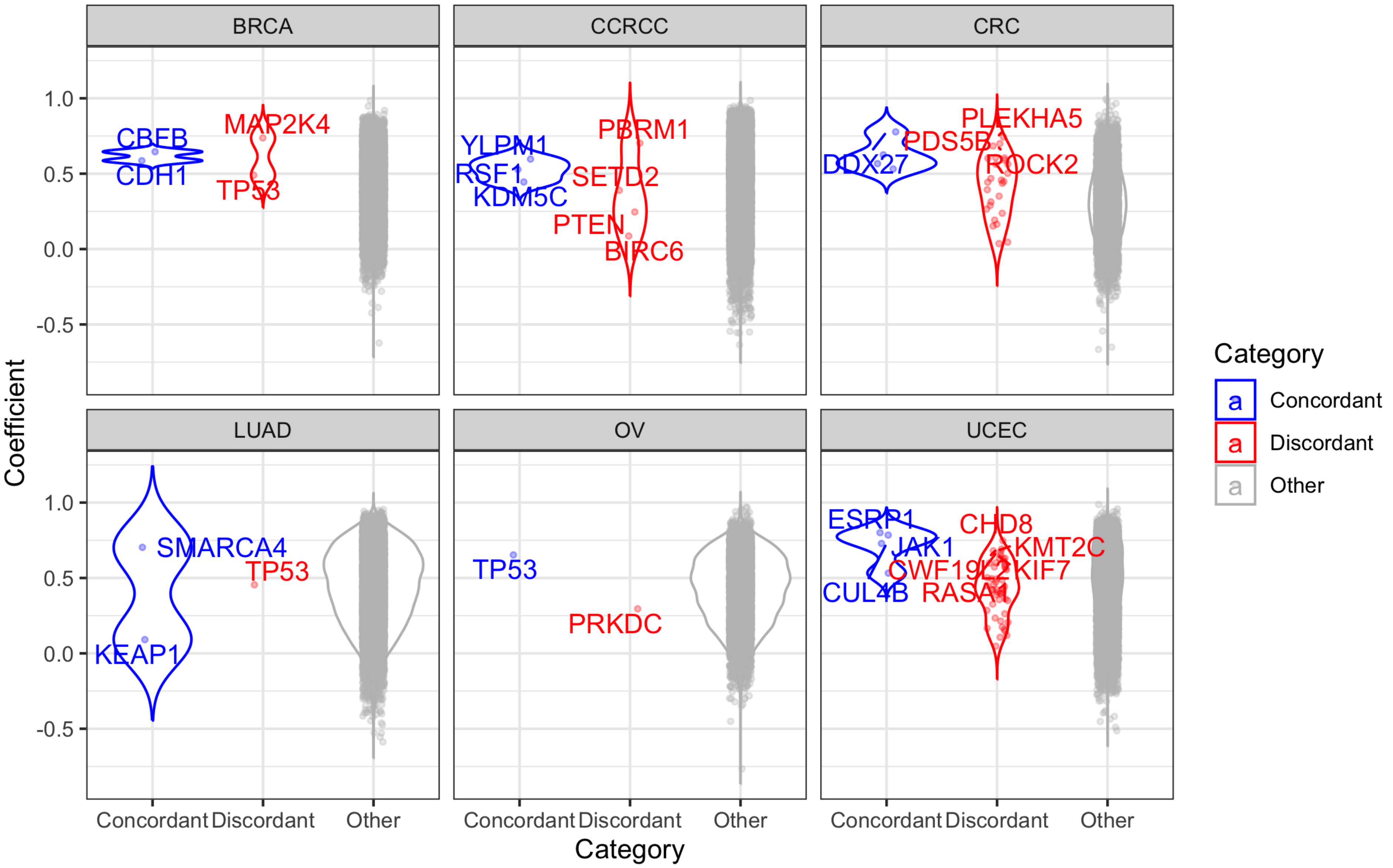

BRCA
CCRCC
CRC
LUAD
OV
UCEC
Coefficient
Category
1.0
0.5
0.0
-0.5
Concordant
Discordant
Other
CBFB
CDH1
MAP2K4
TP53
YLPM1
RSF1
KDM5C
PBRM1
SETD2
PTEN
BIRC6
DDX27
PLEKHA5
PDS5B
ROCK2
SMARCA4
KEAP1
TP53
TP53
PRKDC
ESRP1
JAK1
CUL4B
CHD8
KMT2C
CWF19L2
KIF7
RASA1
Category
a Concordant
a Discordant
a Other
