# Supplementary material for: Mutation impact on mRNA versus protein expression across human cancers
Source: Gigascience. 2025 Jan 6;14:giae113. doi: 10.1093/gigascience/giae113 (PMC11702362; doi:10.1093/gigascience/giae113)
Supplement: giae113_GIGA-D-24-00168_Revision_2 [file giae113_giga-d-24-00168_revision_2.pdf]

GigaScience

Mutation Impact on mRNA Versus Protein Expression across Human Cancers

--Manuscript Draft--

**Manuscript Number:**	GIGA-D-24-00168R2
**Full Title:**	Mutation Impact on mRNA Versus Protein Expression across Human Cancers
**Article Type:**	Research
**Funding Information:**	National Institute of General Medical Sciences (R35GM138113) — Dr Kuan-lin Huang<br>American Cancer Society (RSG-22-115-01-DMC) — Dr Kuan-lin Huang
**Abstract:**	Background<br>Cancer mutations are often assumed to alter proteins, thus promoting tumorigenesis. However, how mutations affect protein expression—in addition to gene expression—has rarely been systematically investigated. This is significant as mRNA and protein levels frequently show only moderate correlation, driven by factors such as translation efficiency and protein degradation. Proteogenomic datasets from large tumor cohorts provide an opportunity to systematically analyze the effects of somatic mutations on mRNA and protein abundance and identify mutations with distinct impacts on these molecular levels.<br><br>Results<br>We conduct a comprehensive analysis of mutation impacts on mRNA- and protein-level expressions of 953 cancer cases with paired genomics and global proteomic profiling across six cancer types. Protein-level impacts are validated for 47.2% of the somatic expression quantitative trait loci (seQTLs), including CDH1 and MSH3 truncations, as well as other mutations from likely “long-tail” driver genes. Devising a statistical pipeline for identifying somatic protein-specific QTLs (spsQTLs), we reveal several gene mutations, including NF1 and MAP2K4 truncations and TP53 missenses showing disproportional influence on protein abundance not readily explained by transcriptomics. Cross-validating with data from massively parallel assays of variant effects (MAVE), TP53 missenses associated with high tumor TP53 proteins are more likely to be experimentally confirmed as functional.<br><br>Conclusion<br>This study reveals that somatic mutations can exhibit distinct impacts on mRNA and protein levels, underscoring the necessity of integrating proteogenomic data to comprehensively identify functionally significant cancer mutations. These insights provide a framework for prioritizing mutations for further functional validation and therapeutic targeting.
**Corresponding Author:**	Kuan-lin Huang, PhD<br>Icahn School of Medicine at Mount Sinai<br>New York, NY UNITED STATES
**Corresponding Author Secondary Information:**	
**Corresponding Author's Institution:**	Icahn School of Medicine at Mount Sinai
**Corresponding Author's Secondary Institution:**	
**First Author:**	Yuqi Liu
**First Author Secondary Information:**	
**Order of Authors:**	Yuqi Liu<br>Abdulkadir Elmas<br>Kuan-lin Huang, PhD

Powered by Editorial Manager® and ProduXion Manager® from Aries Systems Corporation

**Order of Authors Secondary Information:**	
**Response to Reviewers:**	We would like to re-submit our manuscript titled “Mutation Impact on mRNA Versus Protein Expression across Human Cancers” (GIGA-D-24-00168), addressing all the editorial comments:<br><br>1) Please include a citation to your new GigaDB dataset (including the DOI link) to your reference list, and cite this in the data availability section. Completed.<br><br>2) Please structure your abstract ("Background - Results - Conclusions") Completed.<br><br>3) Please submit the manuscript text without embedded figures. Please upload the figures separately in Editorial Manager (one file per figure in good resolution - please refer to the formatting instructions on our homepage). Completed.<br><br>4) Please move URLs to the bibliography and cite them by reference number, rather than including them in the text directly (e.g. line 562, 570, 571). (We treat internet sources as citable objects). Completed.<br><br>5) Please rename the "code availability" section as "Availability of supporting source code and requirements" and use this tabular format: Completed.<br><br>6) For reference numbers in the text, please use square brackets (e.g. [1]) instead of superscript numbers. Completed.<br><br>7) Please also note the reviewer's comment below regarding the equation - although it looks fine in the Word document I'm looking at now, I believe this was just a conversion problem in the PDF. Yes it looked fine on our end, attached also the PDF we converted ourselves which looked fine.<br><br>8) Please also ensure that your revised manuscript conforms to the journal style, which can be found in the Instructions for Authors on the journal homepage Completed.<br><br>Sincerely and on behalf of the team,<br><br>Kuan-lin Huang, Ph.D.<br>Associate Professor of Genetics and Genomic Sciences & Artificial Intelligence and Human Health
**Additional Information:**	
**Question**	**Response**
Are you submitting this manuscript to a special series or article collection?	No
**Experimental design and statistics**<br><br>Full details of the experimental design and statistical methods used should be given in the Methods section, as detailed in our Minimum Standards Reporting Checklist. Information essential to interpreting the data presented should be made available in the figure legends.<br><br>Have you included all the information requested in your manuscript?	Yes

**Resources**<br><br>A description of all resources used, including antibodies, cell lines, animals and software tools, with enough information to allow them to be uniquely identified, should be included in the Methods section. Authors are strongly encouraged to cite Research Resource Identifiers (RRIDs) for antibodies, model organisms and tools, where possible.<br><br>Have you included the information requested as detailed in our Minimum Standards Reporting Checklist?	Yes
**Availability of data and materials**<br><br>All datasets and code on which the conclusions of the paper rely must be either included in your submission or deposited in publicly available repositories (where available and ethically appropriate), referencing such data using a unique identifier in the references and in the "Availability of Data and Materials" section of your manuscript.<br><br>Have you have met the above requirement as detailed in our Minimum Standards Reporting Checklist?	Yes

Powered by Editorial Manager® and ProduXion Manager® from Aries Systems Corporation

Manuscript

Click here to access/download;Manuscript;eQTLmanuscript_for_production.d

Mutation Impact on mRNA Versus Protein Expression across Human Cancers

Yuqi Liu[1*], Abdulkadir Elmas[1*], Kuan-lin Huang[1#]

[1] Department of Genetics and Genomic Sciences, Department of Artificial Intelligence and Human Health, Center for Transformative Disease Modeling, Tisch Cancer Institute, Icahn Genomics Institute, Icahn School of Medicine at Mount Sinai, New York, NY 10029, USA.

* These authors contributed equally to this work.

ORCID: Yuqi Liu [0009-0006-3177-5417]; Abdulkadir Elmas [0000-0002-7999-5770]; Kuan-lin Huang [0000-0002-5537-5817]

[#]Corresponding Author:
Kuan-lin Huang, Ph.D.
Departments of Genetics and Genomic Sciences & Artificial Intelligence and Human Health
Icahn School of Medicine at Mount Sinai
New York, NY 10029
Email: kuan-lin.huang@mssm.edu

ABSTRACT

Background

Cancer mutations are often assumed to alter proteins, thus promoting tumorigenesis. However, how mutations affect protein expression—in addition to gene expression—has rarely been systematically investigated. This is significant as mRNA and protein levels frequently show only moderate correlation, driven by factors such as translation efficiency and protein degradation. Proteogenomic datasets from large tumor cohorts provide an opportunity to systematically analyze the effects of somatic mutations on mRNA and protein abundance and identify mutations with distinct impacts on these molecular levels.

Results

We conduct a comprehensive analysis of mutation impacts on mRNA- and protein-level expressions of 953 cancer cases with paired genomics and global proteomic profiling across six cancer types. Protein-level impacts are validated for 47.2% of the somatic expression quantitative trait loci (seQTLs), including *CDH1* and *MSH3* truncations, as well as other mutations from likely “long-tail” driver genes. Devising a statistical pipeline for identifying somatic protein-specific QTLs (spsQTLs), we reveal several gene mutations, including *NF1* and *MAP2K4* truncations and *TP53* missenses showing disproportional influence on protein abundance not readily explained by transcriptomics. Cross-validating with data from massively parallel assays of variant effects (MAVE), *TP53* missenses associated with high tumor TP53 proteins are more likely to be experimentally confirmed as functional.

Conclusion

This study reveals that somatic mutations can exhibit distinct impacts on mRNA and protein levels, underscoring the necessity of integrating proteogenomic data to comprehensively identify functionally significant cancer mutations. These insights provide a framework for prioritizing mutations for further functional validation and therapeutic targeting.

INTRODUCTION

Cancer arises from the acquisition of mutations that confer selective advantages. The majority of these mutations are thought to affect cellular functions by regulating the expression of gene products. For example, truncations can result in nonsense-mediated decay (NMD)[1], [2], which protects eukaryotic cells through degrading premature termination codon (PTC) bearing mRNA[3]. Additionally, a fraction of cancer mutations may uniquely affect protein abundance but not mRNA expression. However, previous studies characterizing genomic mutations affecting mRNA vs. protein levels have focused on germline variants as expression quantitative trait loci (eQTL)[4], [5], [6]. While other cancer studies have characterized the effect of somatic mutations on mRNA expression levels[7], [8], [9], it remains unclear how somatic mutations may affect protein abundance. The gap of knowledge is critical given that mRNA and protein levels are only moderately correlated[10], [11], [12], [13]. A myriad of factors, including cell state transition, signal delay, translation on demand, and cellular energy constraint, can lead to discrepancies between mRNA and protein levels[14]. Understanding protein-level consequences of cancer mutations is critical in identifying functionally important mutations and revealing their downstream mechanisms.

In recent years, advances in mass spectrometry (MS) technologies have generated a wealth of global proteomic profiles of primary tumor cohorts, many of which also have concurrent genomic and transcriptomic profiling[15], [16], [17], [18], [19], [20]. These proteogenomic datasets present ample opportunities to validate somatic mutations that show concordant impacts on downstream mRNA and protein levels. On the other hand, protein abundance may also be uniquely influenced by the efficiency of protein translation, transport, and degradation. Thus, proteogenomic analyses can reveal mutations that disproportionally impact protein abundances that may not be found using genomic analyses alone.

Herein, we conducted a systematic analysis to decode the relationship between somatic mutations vs. mRNA and protein levels using data from nearly a thousand cases across six cancer types in prospective and retrospective cohorts from the Clinical Proteomic Tumor Analysis Consortium (CPTAC). We identified mutations showing concordant

effects at both mRNA and protein expression levels *in cis*, as well as those that showed protein-specific effects. We further examined how mutations associated with expression changes may predict *in vitro* and *in vivo* functional effects measured by a massively parallel assays of variant effects (MAVE) of TP53[21]. Our results highlight the importance of pairing genomic and proteomic analyses to prioritize functionally important mutations.

RESULTS

Mutation impacts on the mRNA and protein levels

Following the study workflow (**Figure 1A**), we first sought to identify somatic mutations that may impact the corresponding gene's mRNA expression (somatic eQTL, termed seQTL below) and protein abundance (somatic pQTL, termed spQTL below) in primary tumor tissue samples. We performed a multiple regression analysis adjusted for age, gender, ethnicity, and TMT batch using the prospective CPTAC datasets that included matched DNA-Seq, RNA-Seq, and mass spectrometry (MS) global proteomics data of primary tumor samples across six cancer types (see **Methods**, **Figure 1B**), including 115 breast cancer (BRCA)[19], 95 colorectal cancer (CRC)[16], 110 clear cell renal cell carcinoma (CCRCC)[15], 109 lung adenocarcinoma (LUAD)[17], 84 ovarian cancer (OV)[20], and 97 uterine corpus endometrial carcinoma (UCEC)[18], as well as proteogenomic datasets for additional, retrospective BRCA[11], CRC[13], and OV[12] cohorts from CPTAC for validation (**Figure S1A**). We focused on coding mutations given the coverage of the whole-exome sequencing (WES) data used in CPTAC studies; the analyses were further stratified for truncations, missense, and synonymous mutations given their likely different mechanisms of action in affecting levels of the mutated gene product.

Based on the statistical power achieved by these cohort sizes and to reduce false positives, we focused on genes with three or more samples affected by mutations in each functional class of missense, truncation, and synonymous within the cancer cohort, including 134, 13, and 15 genes tested in BRCA; 1360, 318, and 226 genes tested in CRC; 55, 12, and 4 genes tested in CCRCC; 94, 4, and 8 genes tested in LUAD; 134, 5, and 8 genes tested in OV; 2243, 273, and 196 genes tested in UCEC. We sought to

identify their seQTLs affecting *cis*-expression, i.e., expression of the mutation-affected genes. Using the multiple regression model (see **Methods**), we identified 74 gene-cancer seQTL pairs (FDR < 0.05), including 4 in BRCA, 47 in CRC, 7 in CCRCC, 3 in LUAD, 1 in OV, and 12 in UCEC (**Figure 2A, Table S1**). Separated by the functional classes of mutations, 22 of those seQTLs are missense mutations, 12 are synonymous, and 40 are truncating. Top seQTLs showing up-regulation of gene expression are primarily missenses, including *SMARCA4* in LUAD, *WNT7B* in CRC, *TP53* in OV, and *FOXR2* in UCEC. Top candidates showing down-regulation of gene expression include *TP53* and *CDH1* truncations in BRCA, as well as *TP53* truncations in OV (**Figure 2B**).

Using a similar multiple regression but modeling protein abundance as the dependent variable, we identified 103 significant gene-cancer spQTL pairs (FDR < 0.05), including 4 in BRCA, 31 in CRC, 8 in CCRCC, 3 in LUAD, 2 in OV, and 55 in UCEC (**Figure 2A, Table S2**). Compared to the proportion of gene-mutation type evaluated in each cancer type, spQTLs showed significant enrichment for truncations (Fisher exact test p-value < 0.05; **Figure 2A**), highlighting the persistent and more profound effect of truncations on protein abundance compared to mRNA levels. Among the identified spQTLs across cancer, 7 are missense and 96 are truncating. For example, truncating mutations of *NF1* and *ARID1A* in UCEC, and *YLPM1* in CCRCC are each associated with reduced protein level of the corresponding gene (**Figure 2B**). Notably, *TP53* missenses in OV, BRCA, LUAD, and UCEC are each significantly associated with increased protein expression in mutation carriers (**Figure 2B**).

To verify these discoveries, we applied the same seQTL and spQTL analyses using retrospective CPTAC data (**Figure S1A**) that included independent cohorts of BRCA[11], CRC[13], and OV[12] primary tumors. While these cohorts afforded smaller sample sizes, 8 seQTLs and 5 spQTLs were detected in both retrospective and prospective sets. The gene-cancer spQTL pairs showing strong validation in both datasets include *TP53* missense mutations and *CDH1* truncations in BRCA, and *TP53* truncations in CRC (**Figure S1B**).

Mutations showing concordant effects at mRNA and protein levels

We next examined the concordance of seQTL and spQTL associations for each gene-cancer type pair. As expected, for most (88.9%) of the significant seQTLs whose genes had sufficient observations at both the mRNA and protein levels, the identified associations showed the same directionality. However, we only identified 17 seQTLs (47.2%) that are also significant spQTLs at an FDR < 0.05, which we show as concordant QTLs (**Figure 3A, Table S3**). The effect sizes (in log fold change) of these gene-cancer pairs showing concordant seQTLs and spQTLs showed a high correlation between mRNA and protein (Pearson r = 0.90, p-value < 7.51E-7).

In different cancer types, genes whose mutation impacts on gene and protein expressions are concordant include well-known drivers of the disease, including *TP53* missense mutations in OV, *CDH1* truncations in BRCA, and *MSH3* truncations in CRC. Up-regulation of mutated *TP53* in OV is the only association found for genes affected by missense mutations. The 16 other concordant se/spQTLs are all truncations associated with reduced expression and highlight some "long-tail" driver genes, including *PBRM1* in CCRCC, *YLPM1* in CCRCC/UCEC, and *ESRP1* in UCEC (**Figure 3B**). The concordant QTLs with truncating mutation can likely be explained by NMD, which reduces gene expression and in turn diminishes the expression of the corresponding proteins[3]. Compared to the substantially higher counts of seQTL associations (**Figure 2A-B**), these concordant se/spQTL effects validate mutation impacts on the gene product.

Protein-specific mutation impacts not observed at mRNA levels

While most seQTLs and spQTLs show concordance, we postulate that certain mutations may uniquely affect protein abundance but not mRNA levels, which we term somatic protein-specific QTLs (spsQTLs). To identify spsQTLs, we applied two methods to stringently retain QTLs with discordant effects at mRNA and protein levels. First, applying a likelihood ratio test (LRT) between two regression models of protein level being predicted by mRNA level with or without the mutation term (see **Methods**)[4], 96 candidate spsQTLs (FDR < 0.05) were identified. Second, complementing this LRT test

with an approach filtering for gene-cancer pair showing significant spQTL (FDR < 0.05) but not seQTLs (see **Methods**) [22], 86 candidate spsQTLs (FDR < 0.05) were identified.

By overlapping candidate spsQTLs identified by both methods, we retained 83 spsQTLs, the majority (92.8%) of which are truncating mutations (**Figure 4A, Table S4**). Top spsQTLs associated with diminished protein expression include *NF1* truncations in UCEC, *PLEAHK5* truncations in CRC, and *MAP2K4* truncations in BRCA. The only spsQTLs that increase protein expression include *TP53* missense mutations in BRCA, LUAD, and UCEC. (**Figure 4B**). We further examined the discordance in mutation impacts on gene and protein expression levels (**Figure 4C**). While some of these truncations, such as *NF1* in UCEC and *MAP2K4* in BRCA, were often accompanied by lower-than-median mRNA expression in their respective tumor cohorts, their impacts were strikingly observed at diminished protein expression levels. We highlighted in **Figure S2A** spsQTLs where the affected gene's protein showed negative protein log fold-change (logFC) whereas the mRNA logFC is non-negative, including *CASP8* truncations in UCEC, *ARID1A* truncations in CRC and UCEC, and ATM truncations in LUAD and UCED. We also identified a set of spsQTLs truncations, where the logFC associated with a reduction in proteins is 15 times greater than mRNAs logFC (**Figure S2B**). These results suggest that NMD associated with these gene truncations are closely tied to the terminated translation but may not affect mRNA expression to the same degree [23].

To complement the cross-tumor analyses, we also utilized the CPTAC samples with paired tumor-normal tissues to conduct paired differential expression tests for both protein and mRNA expression (**Figure 1A**). The paired sample sizes with proteomic data include 17 in BRCA, 17 in UCEC, 84 in CCRCC, 100 in LUAD, 29 in CRC, and 10 in OV (**Figure 1B**). Covariates including age at diagnosis, ethnicity, race, and sequencing operator are adjusted in the analysis. While this analysis had varied statistical power due to different normal tissue availabilities across cancer types, it served as an independent validation of spQTLs (**Table S5**). This paired tumor-normal analysis validated the protein-level impacts of several discordant spsQTLs (**Figure S3A**) as well as some concordant se/spQTLs (**Figure S3B**). For example, the validated discordant spsQTLs include truncations of

SMAD4 and *SCRIB* in CRC as well as *NF1, GLYR1,* and *RASA1* in UCEC (**Figure S3A**). The validated concordant se/spQTLs include truncations of *YLPM1 and PBRM1* in CCRCC, *SMARCA4* and *KEAP1* in LUAD, and *ESRP1* as well as *JAK2* in UCEC (**Figure S3B**).

Functional evidence of *TP53* missenses associated with high protein expression

Notably, *TP53* missenses are associated with higher protein expression in multiple cancer cohorts, in addition to the expected reduction in expression associated with truncations (**Figure 5A**). Such cis-effect of functional *TP53* missense mutations had previously been observed through immunohistochemistry (IHC[24]) or MS global proteomics experiments[25]. Here, we hypothesized that functional *TP53* missense mutations are more likely to show high levels of concurrent protein-level expression in the mutated tumor sample. To test this hypothesis, we compared gene and protein-level *TP53* expression from CPTAC with *TP53* mutation-level functional data from the *in vitro* and *in vivo* MAVE experiment conducted by Kotler et al[21], where they designed a p53 variants library to study the functional impact of those mutations.

We divided the *TP53* missense mutations from Kotler et al. into three categories: (1) *TP53* mutations with top 20% mRNA or protein expression in the prospective CPTAC cohorts, (2) the other *TP53* mutations observed across all CPTAC samples, and (3) the rest of the assayed *TP53* mutations from Kotler et al. For *in vitro* data, the number of tested mutations by each category is 32, 78, and 1,033, respectively. For *in vivo* data, the number of tested mutations by each category is 19, 10, and 381, respectively. We first compared the relative fitness score (RFS) measured from the *in vitro* assays[17]. While there may be a trend, we did not observe a significant difference between all the other mutations versus *TP53* missenses associated with either top 20% expression based on either mRNA (p-value = 0.090, Wilcoxon rank-sum test) or protein expression (p-value = 0.720).

We next compared the *in vivo* enrichment scores across the same categories, and found that *TP53* missenses associated with top 20% protein expression showed significantly

higher enrichment score *in vivo* compared to that of other *TP53* missenses found in CPTAC (p-value = 0.016) or other experimentally-measured *TP53* mutations (p-value = 3.23E-5, **Figure 5B, Table S6**). In comparison, *TP53* missenses associated with top 20% mRNA expression did not show a significant *in vivo* score difference to that of other *TP53* missenses found in CPTAC (p-value = 0.170). Kotler et al. observed that there was no significant correlation between enrichment score *in vivo* and RFS *in vitro*, which is consistent with our observations and may be explained by the different selective pressures between these settings *in vivo* and *in vitro*[21]. Finally, *TP53* missenses associated with top 20% protein expression (p-value = 5.91E-7) or top 20% mRNA expression (p-value = 2.38E-2) showed significantly higher prevalence than other CPTAC mutations based on counts from the International Agency for Research on Cancer (IARC) database[21] (**Figure 5B, Table S6**). Overall, these analyses suggested that protein-level consequences from primary tumor samples can aid the identification of functional mutations.

DISCUSSION

Herein, we analyzed how somatic mutations affect mRNA and protein levels using matched genomic, transcriptomic, and global proteomic data from 953 cases across six solid cancer types. We first investigated the mutation impacts at the mRNA level and protein level, finding that although most seQTLs have the same direction of effect as spQTLs, less than half of them are also significant at the protein level. We also studied the concordant or discordant relationship between seQTL versus spQTLs, finding several spsQTLs that have disproportional effects on protein. Finally, we conducted analyses to provide functional validation[21] for our findings of TP53 missenses associated with high protein expression.

Integrating protein-level data identified nearly 47.2% seQTLs as concordant, significant spQTLs. The result demonstrates the capacity of proteomic data to validate genomic findings and potentially filter out noises that may arise for example due to the more

transient nature of transcription compared to translation. In addition to well-known tumor suppressors like *TP53* and *MSH3*, other gene mutations with concordant effects may also be “long tail” driver genes that will otherwise require large cohort sample sizes to discover. For example, *PBRM1*, which we found in CCRCC, is a subunit of the PBAF chromatin remodeling complex thought to be a tumor suppressor gene whose mutations may confer synthetic lethality to DNA repair inhibitors[26]. *ESRP1*, found in UCEC, is crucial in regulating alternative splicing and the translation of some genes during organogenesis[27]. Other less-studied genes we identified include *YLPM1* truncations associated with concordantly reduced *YLPM1* mRNA and protein expression levels in both CCRCC and UCEC. Analyzing the distribution of these gene mutations on NCI’s Genome Data Commons, we observed many other recurrent truncations (**Figure S4**), suggesting these mutations may represent some of the “long tail” driver mutations that warrant further investigation[28], [29].

By devising a specific pipeline to detect spsQTLs, our results showed that apart from mutations that influence protein level mediated by changes in mRNA level, many mutations are associated with disproportional aberrations at the protein level compared to mRNA changes, indicating post-transcriptional regulation. SpsQTLs were found to affect known driver genes such as *TP53* missenses, and truncations in *NF1*[30] and *MAP2K4*[31]. In most cases, protein molecules are more direct mediators of cellular functions and phenotypes than mRNAs[32]. Thus, the discordant effect between mRNA level and protein level discovered in our study highlights the importance of exploring disease mechanisms and developing treatments at the protein level.

One possible source of spsQTLs is the imperfect correlation between mRNA and protein expression in the affected genes. Additional statistical analyses revealed that this mRNA-protein correlations range widely across genes and cancer types (**Figure S5**). While genes harboring spsQTLs have lower mRNA-protein correlations in general than genes with concordant eQTL and pQTL, this is not the case for several discordant genes, including MAP2K4 in BRCA and PBRM1 in CCRCC (**Table S7**). Based on the number of mutations and genes identified, CRC and UCEC reached statistically significant

differences between concordant and all other expressed genes (Wilcoxon rank-sum tests, $p = 0.0056$ and $p = 0.022$, respectively); in CRC, mRNA-protein correlations also showed significant differences between discordant and all other expressed genes ($p = 0.013$ and $p = 0.29$, respectively); other cancer types likely did not reach statistical significance likely due to sufficient mutations identified. The imperfect correspondence between gene mRNA-protein correlations and mutation impacts further stresses the need to analyze and consider protein-specific impacts of mutations. **Table S7** provides complete mRNA-protein correlation data for all concordant/discordant eQTL/pQTLs in their respective cancer type for in-depth examination.

This study has several limitations. First, our findings do not distinguish between several potential mechanisms that could lead to discordant effects of mutations on gene and protein expression. One possibility is that the mutation affects the efficiency of translation, leading to changes in protein levels that are not reflected in mRNA levels. For example, accumulating evidence in recent years suggests that NMD is closely tied to the termination of translation[23], which may explain instances where some truncations afford much stronger associations with protein levels in our findings. But, in many cases, the mechanisms of how mutations may affect protein abundance may be context- and gene-specific and remain to be elucidated. For example, certain mutations may influence the binding of RNA binding proteins and the efficiency of translation, whereas others may alter post-translational modifications, such as phosphorylation or ubiquitination, which can impact protein stability or degradation without affecting transcription or translation rates. Second, the proteogenomic tumor cohorts used herein, while being some of the largest studies to date, still are limited in sample sizes and preclude sufficient statistical power to identify pQTLs at a single mutation level or reveal *trans* effects. Third, given the limitation of current omic technology and data, our findings do not resolve mutation impact on proteins at the temporal, spatial, or single-cell resolution, but provide candidate mutations to be investigated in future studies. Fourth, our regression models assume a linear relationship between mutations (one gene at a time), confounders, and expression, which may not capture more complex, nonlinear effects of mutations on multiple mRNA or protein expression. Future studies could explore non-linear regression models or

neural network approaches to better account for these effects. Fifth, we employed two complementary methods to confidently identify spsQTLs that represent true protein-specific regulatory events. However, the reliance on FDR thresholds could still limit the detection of spsQTLs with subtle effects. Alternative approaches, such as Bayesian models that account for prior biological knowledge or hierarchical modeling, could be considered in future analyses to improve the specificity of spsQTL detection. Additionally, while our method focuses on cis-acting mutations, potential trans-acting effects could be missed, a limitation that should be explored in larger datasets or by incorporating network-based analyses.

Finally, using *TP53* missense mutations as an example, we showed that protein-level expression can serve as an effective strategy to prioritize functional mutations. As DNA-Seq become ever more commonplace, many rare mutations are being identified and it remains challenging to accurately classify their functional impacts. Our data demonstrated that *TP53* missenses associated with high protein expression show significantly higher functional scores, particularly those measured *in vivo*. This protein-expression-based prioritization strategy can be particularly powerful when combined with high-throughput functional assays like using MAVE model systems that are typically *in vitro*. Considering that both MAVE and proteogenomic datasets of tumor cohorts are both expanding quickly in the next few years[33], [34], the combined approaches can help effectively pinpoint functional mutations for mechanistic and clinical characterization. The prioritized mutations based on protein-level consequences may also guide the selection of targeted therapy to advance precision medicine.

METHODS

Proteogenomic datasets

The prospective CPTAC data were downloaded and processed as described in the Method section of the work of Elmas et al. [35]. The overview table in **Figure 1A** of the dataset describes, for each cancer cohort, the sample size, female patient percentage, average cancer onset age, and tumor stage. Samples are normalized by their median absolute deviations (MAD), so that the MAD of all samples in the dataset is 1. Protein

markers with high fractions (greater than 20%) of missing values are filtered out. For the corresponding RNA-seq data, we used the log2 normalization on the FPKM (fragments per kilobase of exon per million mapped fragments)-normalized RNA-seq counts and genes that have no expression in at least 90% of the samples were filtered out.

The proteomics data used for validation were downloaded from the NCI CPTAC portal [36]. The dataset overview table in **Figure S1A** describe for each cancer cohort the sample size, female patient percentage, average cancer onset age, and tumor stage. The validation data are processed in the same way as the prospective data. The RNA-seq data sets of the three retrospective CPTAC cohorts were downloaded from the NCI CPTAC DCC portal[36]. The RNA expression was measured in Fragments Per Kilobase of transcript per Million mapped reads (FPKM)[37][38] and was further normalized by log2(FPKM+1).

pQTL and eQTL identification

For each cancer cohort, we identified pQTLs and eQTLs using the multiple linear regression model as implemented in the “limma” R package (v3.42.2)[39]. We also corrected confounding factors including age, gender, ethnicity, and TMT batch. The false discovery rate (FDR) was corrected from the p-values with the Benjamini-Hochberg procedure[40], ensuring that the identified QTLs are statistically robust. Somatic mutations are grouped at a gene level in the multiple regression model, similar to that implemented by our previously developed AeQTL tool[7]. Mutations are separately analyzed by their mechanisms of action, including nonsynonymous mutations that likely do not affect expression, missense mutations, and truncating mutations—including frameshift and in-frame indels, nonsense, splice site, and translation start site mutations. To improve statistical power, we focused our analysis on genes with three or more mutations in each cancer cohort and analyzed associations of mutations affecting *cis*-expression of the corresponding mRNA or protein products.

spsQTL identification

We combined two complementary statistical methods to identify spsQTLs. In the first method adopted from Battle et al.[4], we compared the following two nested linear models using likelihood ratio test (LRT) with the “anova” function in R:

$$\square = \square + \square_{0}\square + \square_{1}\square$$
$$\square = \square + \square_{2}\square$$

where $\square$ is the genotype, $\square$ represents RNA level, and p is the protein level. By comparing these models using LRT and filtering results with an FDR less than 0.05, we identified candidate spsQTLs where the genotype (mutation) has a disproportionate impact on protein abundance independent of mRNA expression.

In the second method adopted from Mirauta et al.[22], we selected QTLs where the spQTL FDR was less than 0.05 but the corresponding seQTL FDR was greater than 0.05 as candidate spsQTLs, to specifically identify mutations that affect protein levels without influencing mRNA. We then overlapped these two lists of candidate spsQTLs obtained from two complementary methods to identify the final list of spsQTLs for downstream analyses.

mRNA-Protein correlation:

To investigate the impact of mutations on mRNA and protein expression, we performed a comparative analysis across the six solid cancer types. For each cancer type, Pearson correlation coefficients were calculated for individual genes using paired mRNA and protein expression data. We analyzed three groups of genes we identified as showing variable impact on mRNA/protein level expressions: Concordant genes (with mutations showing concordant effects at both mRNA and protein levels in cis), Discordant genes (showing protein-specific effects), and Other genes (showing no concordant or protein-specific impact). Our aim was to test the hypothesis whether the mRNA-protein correlations of the Concordant/Discordant groups differed from the baseline genome-wide mRNA-protein correlations, indicating biological significance. To assess this, we employed two-sample Wilcoxon rank-sum test, comparing the mRNA-protein correlations for the Concordant/Discordant and Other gene groups within each cancer type. Pairwise comparisons were made between the Concordant and Other gene sets, as well as

between the Discordant and Other gene sets, demonstrating that the correlation coefficients for these groups were drawn from distinct population distributions with statistical significance at a p-value threshold of 0.05.

Tumor-normal differential expression analysis

We conducted this analysis in the prospective CPTAC cohorts with paired tumor-adjacent tissure normal samples. For each cancer cohort, we paired the tumor and normal samples from the same patient and performed a differential protein/mRNA expression analysis to identify differentially expressed proteins with “limma” package. Demographic factors and batch effects, including age, ethnicity, race, and sequencing operator are adjusted in the multiple regression model.

Figures

Figure 1. Overview of the study workflow and proteogenomic cohorts. (A) Study workflow to identify eQTLs, pQTLs, concordant QTLs (between mRNA and protein levels), and spsQTLs showing disproportional effects on protein expression. (B) Summary of the prospective CPTAC proteogenomic cohorts used for the discovery analyses, including cancer type abbreviation, data source, sample size of tumor (T) and normal (N) tissues, female percentage, average onset age in years, and tumor stage distribution.

Figure 2. Gene mutations identified as *cis* seQTLs and spQTLs across six adult cancer types. (A) Overview of the somatic mutation QTLs identified in different cancer types and mutation types, including missense (green), truncating (orange), and synonymous (purple) mutations. For both eQTLs and pQTLs, the panel on the left shows the counts of the mutation-gene pairs included in analyses, and the figure on the right shows the counts of the significant eQTLs and pQTLs. (B) Volcano plots showing seQTLs associations in the six cancer types (left) and volcano plots showing spQTLs associations (right), where each dot denotes a gene-cancer pair included in the analysis. Top associated genes were further labeled. FC: mRNA/protein expression log fold change. FDR: false discovery rate.

Figure 3. Gene mutations showing concordant impacts on gene and protein expression levels. (A) Overview of concordant QTLs as shown by their effect sizes in log[Fold Change (FC)], where the gray line shows when the protein logFC equals RNA logFC. Some of the top concordant QTLs were further labeled by cancer type and gene name. (B) Examples of QTL with concordant effects at mRNA and protein expression levels. For each gene, the plot on the left shows the corresponding mRNA levels of mutation carriers vs. non-carriers in FPKM, and the plot on the right shows protein level comparison in log ratio (MS TMT measurements) in the respective cancer type labeled on top of each of the violin plots. The labeled mutations are the three mutations whose carriers show the highest absolute expression differences of the mutated gene product compared to the non-carriers.

Figure 4. Gene mutations showing discordant impacts on gene and protein expression levels. (A) Overview of discordant QTLs identified by our statistical pipeline as shown by their effect sizes in log[Fold Change (FC)], where the gray line shows when the protein logFC equals RNA logFC. (B) Heatmaps of QTLs that are significant as either seQTL or spQTL and that are shared across at least two cancer types. Brown box indicates significant spsQTLs, and color indicates the effect size in log[Fold Change (FC)], average protein expression of mutation carriers in log ratio from the MS TMT quantifications. (C) Examples of QTL with discordant effects at mRNA vs. protein levels. For each gene, the plot on the left shows the corresponding mRNA levels of mutation carriers vs. non-carriers in FPKM, and the plot on the right shows protein level comparison in log ratio (MS TMT measurements) in the respective cancer type labeled on top of each of the violin plots. The labeled mutations are the three mutations whose carriers show the highest absolute expression differences of the mutated gene product compared to the non-carriers.

Figure 5. Functional verification of *TP53* mutation associated with high mRNA or protein levels using *in vitro* and *in vivo* data from a MAVE experiment. (A) Percentile of averaged expression associated with a given *TP53* mutation at the mRNA (x-axis) and

protein (y-axis) levels in the respective cancer cohort. *TP53* mutations are color coded by mutation type (left) and observed cancer type (right), respectively. (B) Violin plots comparing the in vitro functional score (RFS, top), in vivo enrichment score (middle), and IARC occurrences (bottom) for TP53 mutations in the three groups defined by (1) *TP53* mutations with top 20% mRNA (left) or protein (right) expression in the prospective CPTAC cohorts, (2) the other *TP53* mutations observed across all CPTAC samples, and (3) the rest of the assayed *TP53* mutations from Kotler et al[21].

Supplementary Tables

Table S1. List of expression quantitative trait loci (eQTLs) identified across 6 cancer types. This table provides details on the gene mutations associated with mRNA expression levels, including statistical test results, mutation type, p-values (adjusted), and effect sizes.

Table S2. List of protein quantitative trait loci (pQTLs) identified across 6 cancer types. This table provides details on the gene mutations associated with protein abundance levels, including statistical test results, mutation type, p-values (adjusted), and effect sizes.

Table S3. Concordant expression and protein quantitative trait loci (eQTLs and pQTLs) identified across 6 cancer types. This table includes information on the gene mutations, identified cancer types, and their impact on both mRNA and protein expression levels, demonstrating loci with consistent effects across both molecular layers.

Table S4. Significant somatic protein-specific QTLs (spsQTLs) identified by our statistical pipeline across six cancer types. This table details the loci with mutations showing significant impacts on protein abundance not explained by mRNA levels, including summary statistics for eQTL/pQTL tests and the LRT and overlap test results.

Table S5. Summary statistics for differentially expressed proteins (DEPs) identified in paired tumor-normal (TN) samples across six cancer types. This table includes the test statistics of protein expression differences between tumor and normal tissues harboring the specific mutation.

Table S6. Test statistics between the three groups of TP53 mutations. The tested groups were defined by (1) TP53 mutations with top 20% mRNA (left) or protein (right) expression in the prospective CPTAC cohorts, (2) the other TP53 mutations observed across all CPTAC samples, and (3) the rest of the assayed TP53 mutations from Kotler et al. using *TP53* functional scores form Kotler et al.

Table S7. Pearson's correlation coefficient tests between paired mRNA and protein expressions for each concordant and discordant gene, within each cancer cohort.

Supplementary Figures

Supplementary Figure 1. Overview of the retrospective cohorts (A) Summary of the retrospective CPTAC proteogenomic cohorts used for the discovery analyses, including cancer type abbreviation, data source, sample size of tumor (T) and normal (N) tissues, female percentage, average onset age in years, and tumor stage distribution. (B) Volcano plots showing seQTLs associations in the six cancer types (left) and volcano plots showing spQTLs associations (right), where each dot denotes a gene-cancer pair included in the analysis. Top associated genes were further labeled. FC: log fold change. FDR: false discovery rate.

Supplementary Figure 2. spsQTLs with strong effects. (A) Examples of spsQTL whose effect sizes in mRNA level and protein level are in different direction. For each gene, the plot on the left shows the corresponding mRNA levels of mutation carriers vs. non-carriers in FPKM, and the plot on the right shows protein level comparison in log ratio (MS TMT measurements) in the respective cancer type labeled on top of each of the violin plots. The labeled mutations are the three mutations whose carriers show the highest absolute expression differences of the mutated gene product compared to the non-carriers. (B) Examples of spsQTL with a protein logFC and mRNA logFC ratio greater than 15

Supplementary Figure 3. Overlapped of significant QTLs in cross-tumor analysis and matched tumor-normal analysis projected onto pQTL volcano plots based on cross-tumor analyses. The plots were made separately for (A) discordant spsQTLs, and (B) concordant eQTL/pQTLs.

Supplementary Figure 4. Example lolliplots showing mutations for two genes that were identified as spsQTLs, including YLPM1 and ESRP1. The number on each disc denotes the number of mutations in that position and the color of the disc represents the mutation type.

Supplementary Figure 5. Correlation coefficients of Concordant vs. Discordant genes. The violin plots depict the distribution of correlation coefficients between matched mRNA and protein expressions for Concordant (blue), Discordant (red), and Other genes (gray) across the six cancer types studied. Genes with notable correlations are labeled in each plot.

DATA AND SOFTWARE AVAILABILITY

Data Availability

Proteomic data for CPTAC-2/3 cohorts can be found on National Cancer Institute (NCI) Proteomic Data Commons (PDC) [41]. The studies used in the discovery cohorts and their PDC study IDs are: BRCA (PDC000120), CRC (PDC000116), CCRCC (PDC000127), LUAD (PDC000153), OV (JHU: PDC000110; PNNL: PDC000118), UCEC (PDC000125)

The studies used in the validation cohorts and their PDC study IDs are: BRCA (PDC000173), CRC (PDC000111), OV (JHU: PDC000113; PNNL: PDC000114)

Genomic data, including DNA mutation and transcriptome profiling for all CPTAC-2/3 cohorts used herein can be found on National Cancer Institute (NCI) Genome Data Commons (GDC) [42] (dbGaP Study Accession #: phs000892) and (dbGaP Study Accession #: phs001287) [43].

Data for TP53 MAVE assays can be downloaded from the Supplementary Information from Kotler et al. [21].

Supporting data of our analysis results and an archival copy of the corresponding code are available via the GigaScience repository, GigaDB [44].

Availability of supporting source code and requirements

Project name: Protein expression quantitative trait loci (pQTLs): software and analytic code

Project home page: https://github.com/Huang-lab/pQTL [45]

Operating system(s): Platform independent

Programming language: R, Python, Jupyter Notebook

License: MIT

ACKNOWLEDGEMENTS

The authors wish to acknowledge CPTAC and its participating patients and families that generously contributed the data. This work was supported by NIH NIGMS R35GM138113, ACS RSG-22-115-01-DMC, and Mount Sinai funds to KH.

DECLARATION OF INTERESTS

K.H. is a co-founder and board member of a non-for-profit 501(c)(3) organization, Open Box Science, from which he does not receive any compensation and pose no competing financial interests with this work. All authors declare no competing interests.

CONTRIBUTIONS

K.H. conceived the research; Y.L and K.H. designed the analyses. Y.L. and A.E. developed the software and conducted the bioinformatics analyses, A.E. curated and preprocessed the datasets. Y.L., A.E., and K.H. wrote the manuscript. K.H. supervised the study. All authors read, edited, and approved the manuscript.

REFERENCES

[1] T. Kurosaki, M. W. Popp, and L. E. Maquat, ‘Quality and quantity control of gene expression by nonsense-mediated mRNA decay’, 2019. doi: 10.1038/s41580-019-0126-2.
[2] Z. Wang *et al.*, ‘Non-cancer-related pathogenic germline variants and expression consequences in ten-thousand cancer genomes’, *Genome Med*, vol. 13, no. 1, 2021, doi: 10.1186/s13073-021-00964-1.
[3] R. G. H. Lindeboom, F. Supek, and B. Lehner, ‘The rules and impact of nonsense-mediated mRNA decay in human cancers’, *Nat Genet*, vol. 48, no. 10, 2016, doi: 10.1038/ng.3664.
[4] A. Battle *et al.*, ‘Impact of regulatory variation from RNA to protein’, *Science (1979)*, vol. 347, no. 6222, 2015, doi: 10.1126/science.1260793.
[5] C. Cenik *et al.*, ‘Integrative analysis of RNA, translation, and protein levels reveals distinct regulatory variation across humans’, *Genome Res*, vol. 25, no. 11, 2015, doi: 10.1101/gr.193342.115.
[6] J. M. Chick *et al.*, ‘Defining the consequences of genetic variation on a proteome-wide scale’, *Nature*, vol. 534, no. 7608, 2016, doi: 10.1038/nature18270.
[7] G. Dong, M. C. Wendl, B. Zhang, L. Ding, and K. L. Huang, ‘AeQTL: eQTL analysis using region-based aggregation of rare genomic variants’, *Pac Symp Biocomput*, vol. 26, 2021, doi: 10.1142/9789811232701_0017.
[8] R. Rabadán *et al.*, ‘Identification of relevant genetic alterations in cancer using topological data analysis’, *Nat Commun*, vol. 11, no. 1, 2020, doi: 10.1038/s41467-020-17659-7.
[9] J. Ding *et al.*, ‘Systematic analysis of somatic mutations impacting gene expression in 12 tumour types’, *Nat Commun*, vol. 6, 2015, doi: 10.1038/ncomms9554.
[10] G. Arad and T. Geiger, ‘Functional impact of protein-RNA variation in clinical cancer analyses’, *Molecular & Cellular Proteomics*, p. 100587, Jun. 2023, doi: 10.1016/J.MCPRO.2023.100587.
[11] P. Mertins *et al.*, ‘Proteogenomics connects somatic mutations to signalling in breast cancer’, *Nature*, vol. 534, no. 7605, 2016, doi: 10.1038/nature18003.
[12] H. Zhang *et al.*, ‘Integrated Proteogenomic Characterization of Human High-Grade Serous Ovarian Cancer’, *Cell*, vol. 166, no. 3, 2016, doi: 10.1016/j.cell.2016.05.069.
[13] B. Zhang *et al.*, ‘Proteogenomic characterization of human colon and rectal cancer’, *Nature*, vol. 513, no. 7518, 2014, doi: 10.1038/nature13438.
[14] Y. Liu, A. Beyer, and R. Aebersold, ‘On the Dependency of Cellular Protein Levels on mRNA Abundance’, 2016. doi: 10.1016/j.cell.2016.03.014.
[15] D. J. Clark *et al.*, ‘Integrated Proteogenomic Characterization of Clear Cell Renal Cell Carcinoma’, *Cell*, vol. 179, no. 4, 2019, doi: 10.1016/j.cell.2019.10.007.
[16] S. Vasaikar *et al.*, ‘Proteogenomic Analysis of Human Colon Cancer Reveals New Therapeutic Opportunities’, *Cell*, vol. 177, no. 4, 2019, doi: 10.1016/j.cell.2019.03.030.
[17] M. A. Gillette *et al.*, ‘Proteogenomic Characterization Reveals Therapeutic Vulnerabilities in Lung Adenocarcinoma’, *Cell*, vol. 182, no. 1, 2020, doi: 10.1016/j.cell.2020.06.013.
[18] Y. Dou *et al.*, ‘Proteogenomic Characterization of Endometrial Carcinoma’, *Cell*, vol. 180, no. 4, 2020, doi: 10.1016/j.cell.2020.01.026.

[19] K. Krug *et al.*, ‘Proteogenomic Landscape of Breast Cancer Tumorigenesis and Targeted Therapy’, *Cell*, vol. 183, no. 5, 2020, doi: 10.1016/j.cell.2020.10.036.

[20] J. E. McDermott *et al.*, ‘Proteogenomic Characterization of Ovarian HGSC Implicates Mitotic Kinases, Replication Stress in Observed Chromosomal Instability’, *Cell Rep Med*, vol. 1, no. 1, 2020, doi: 10.1016/j.xcrm.2020.100004.

[21] E. Kotler *et al.*, ‘A Systematic p53 Mutation Library Links Differential Functional Impact to Cancer Mutation Pattern and Evolutionary Conservation’, *Mol Cell*, vol. 71, no. 1, 2018, doi: 10.1016/j.molcel.2018.06.012.

[22] B. A. Mirauta *et al.*, ‘Population-scale proteome variation in human induced pluripotent stem cells’, *Elife*, vol. 9, 2020, doi: 10.7554/ELIFE.57390.

[23] E. D. Karousis and O. Mühlemann, ‘Nonsense-mediated mRNA decay begins where translation ends’, *Cold Spring Harb Perspect Biol*, vol. 11, no. 2, 2019, doi: 10.1101/cshperspect.a032862.

[24] A. M. Davidoff, P. A. Humphrey, J. Dirk Iglehart, and J. R. Marks, ‘Genetic basis for p53 overexpression in human breast cancer’, *Proc Natl Acad Sci U S A*, vol. 88, no. 11, 1991, doi: 10.1073/pnas.88.11.5006.

[25] K. lin Huang *et al.*, ‘Spatially interacting phosphorylation sites and mutations in cancer’, *Nat Commun*, vol. 12, no. 1, 2021, doi: 10.1038/s41467-021-22481-w.

[26] R. M. Chabanon *et al.*, ‘PBRM1 deficiency confers synthetic lethality to DNA repair inhibitors in cancer’, *Cancer Res*, vol. 81, no. 11, 2021, doi: 10.1158/0008-5472.CAN-21-0628.

[27] Y. Vadlamudi, D. K. Dey, and S. C. Kang, ‘Emerging Multi-cancer Regulatory Role of ESRP1: Orchestration of Alternative Splicing to Control EMT’, *Curr Cancer Drug Targets*, vol. 20, no. 9, 2020, doi: 10.2174/1568009620666200621153831.

[28] J. Armenia *et al.*, ‘The long tail of oncogenic drivers in prostate cancer’, *Nat Genet*, vol. 50, no. 5, 2018, doi: 10.1038/s41588-018-0078-z.

[29] S. K. Loganathan *et al.*, ‘Rare driver mutations in head and neck squamous cell carcinomas converge on NOTCH signaling’, *Science*, vol. 367, no. 6483, 2020, doi: 10.1126/science.aax0902.

[30] C. Philpott, H. Tovell, I. M. Frayling, D. N. Cooper, and M. Upadhyaya, ‘The NF1 somatic mutational landscape in sporadic human cancers’, 2017. doi: 10.1186/s40246-017-0109-3.

[31] Z. Xue *et al.*, ‘MAP3K1 and MAP2K4 mutations are associated with sensitivity to MEK inhibitors in multiple cancer models’, *Cell Res*, vol. 28, no. 7, 2018, doi: 10.1038/s41422-018-0044-4.

[32] C. Buccitelli and M. Selbach, ‘mRNAs, proteins and the emerging principles of gene expression control’, 2020. doi: 10.1038/s41576-020-0258-4.

[33] N. J. Edwards *et al.*, ‘The CPTAC data portal: A resource for cancer proteomics research’, *J Proteome Res*, vol. 14, no. 6, 2015, doi: 10.1021/pr501254j.

[34] D. Kuang *et al.*, ‘MaveRegistry: a collaboration platform for multiplexed assays of variant effect’, *Bioinformatics*, vol. 37, no. 19, 2021, doi: 10.1093/bioinformatics/btab215.

[35] A. Elmas, S. Tharakan, S. Jaladanki, M. D. Galsky, T. Liu, and K. lin Huang, ‘Pan-cancer proteogenomic investigations identify post-transcriptional kinase targets’, *Commun Biol*, vol. 4, no. 1, 2021, doi: 10.1038/s42003-021-02636-7.

[36] CPTAC Data Portal https://cptac-data-portal.georgetown.edu/cptac/

[37] National Cancer Institute - GDC documentation: FPKM https://docs.gdc.cancer.gov/Data/Bioinformatics_Pipelines/Expression_mRNA_Pipeline/#fpkm

[38] cpta rna expression (Ding lab) [github repository] https://github.com/ding-lab/cptac_rna_expression'.

[39] M. E. Ritchie *et al.*, 'limma powers differential expression analyses for RNA-sequencing and microarray studies.', *Nucleic Acids Res*, vol. 43, no. 7, p. e47, Apr. 2015, doi: 10.1093/nar/gkv007.

[40] Y. Benjamini and Y. Hochberg, 'Controlling the False Discovery Rate: A Practical and Powerful Approach to Multiple Testing', *J R Stat Soc Series B Stat Methodol*, vol. 57, no. 1, pp. 289–300, Jan. 1995, doi: 10.1111/j.2517-6161.1995.tb02031.x.

[41] National Cancer Institute – Proteomics Data Commons https://cptac-data-portal.georgetown.edu/cptacPublic/

[42] National Cancer Institute – Genomic Data Commons, CPTAC-2 https://portal.gdc.cancer.gov/projects/CPTAC-2

[43] National Cancer Institute – Genomic Data Commons, CPTAC-3 https://portal.gdc.cancer.gov/projects/CPTAC-3'.

[44] Y. Liu, A. Elmas, and K. Huang, 'Supporting data for "Mutation Impact on mRNA Versus Protein Expression across Human Cancers". GigaScience Database. 2024. https://doi.org/10.5524/102598

[45] Protein expression quantitative trait loci (pQTLs): software and analytic code [github repository] 2024. https://github.com/Huang-lab/pQTL'.

Figure 1 Click here to access/download;Figure;fig1.tiff

A

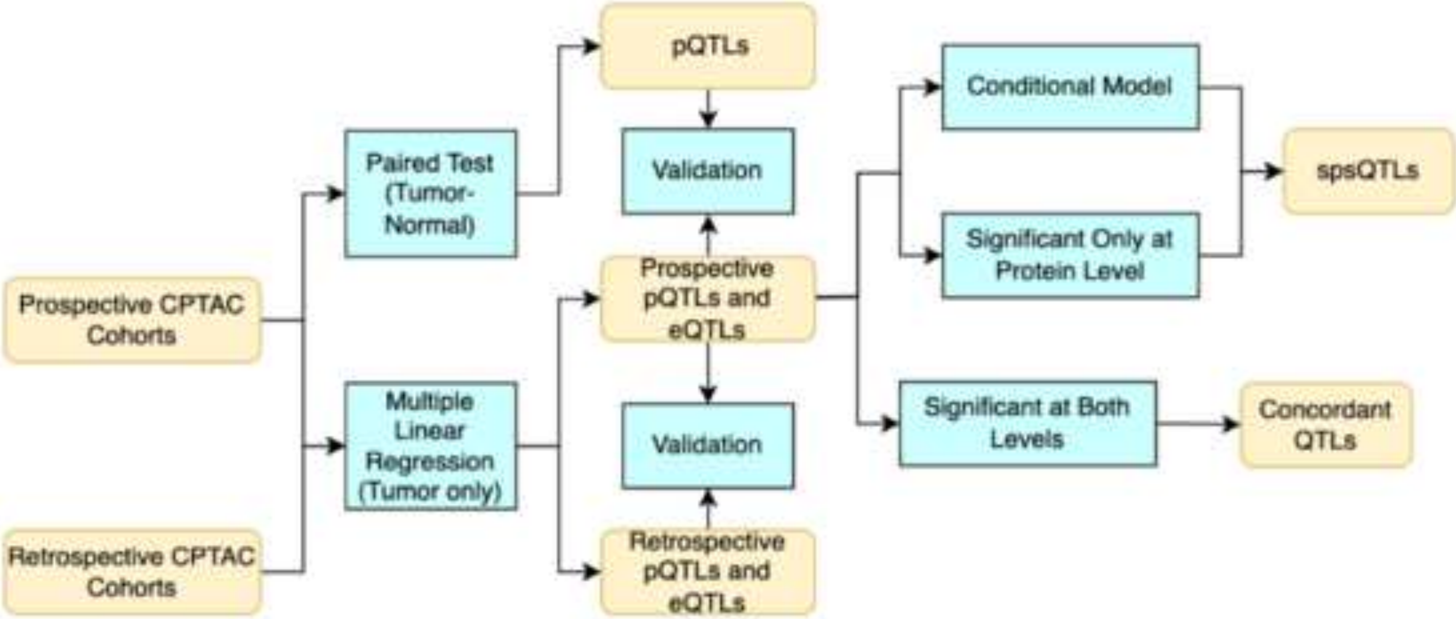

B

Cancer Type	Breast Cancer	Clear Cell Renal Cell Carcinoma	Colorectal Cancer	Lung Adenocarci -noma	Ovarian Cancer	Uterine Corpus Endometrial Carcinoma
Abbreviation	**BRCA**	**CCRCC**	**CRC**	**LUAD**	**OV**	**UCEC**
Data Source	Krug et al. 2020 (PMID: 33212010)	Clark et al. 2019 (PMID: 31675502)	Vasaikar et al. 2019 (PMID: 31031003)	Gillette et al. 2020 (PMID: 32649874)	McDermott et al. 2020 (PMID: 32529193)	Dou et al. 2020 (PMID: 32059776)
Sample Size (Tumors/Normals)	T: 115<br>N: 18	T: 110<br>N: 84	T: 95<br>N: 100	T: 109<br>N: 102	T: 84<br>N: 19	T: 97<br>N: 20
Female %	100%	25.2%	57.4%	34.6%	100%	100%
Average Onset (yr)	60.4	60.6	65.2	62.7	59.1	63.7
Tumor Stage	1: 3%<br>2: 60.4%<br>3: 26.9%<br>NA: 9.7%	1: 41.8%<br>2: 15.5%<br>3: 34.5%<br>4: 8.2%	1: 10.2%<br>2: 40.6%<br>3: 41.1%<br>4: 8.1%	1: 53.5%<br>2: 27.5%<br>3: 18.5%<br>4: 0.5%	1: 1%<br>2: 1%<br>3: 72.8%<br>4: 15.5%<br>NA: 9.7%	1: 76.1%<br>2: 6.8%<br>3: 14.5%<br>4: 2.6%

Figure 2

Click here to access/download;Figure;fig2.tiff

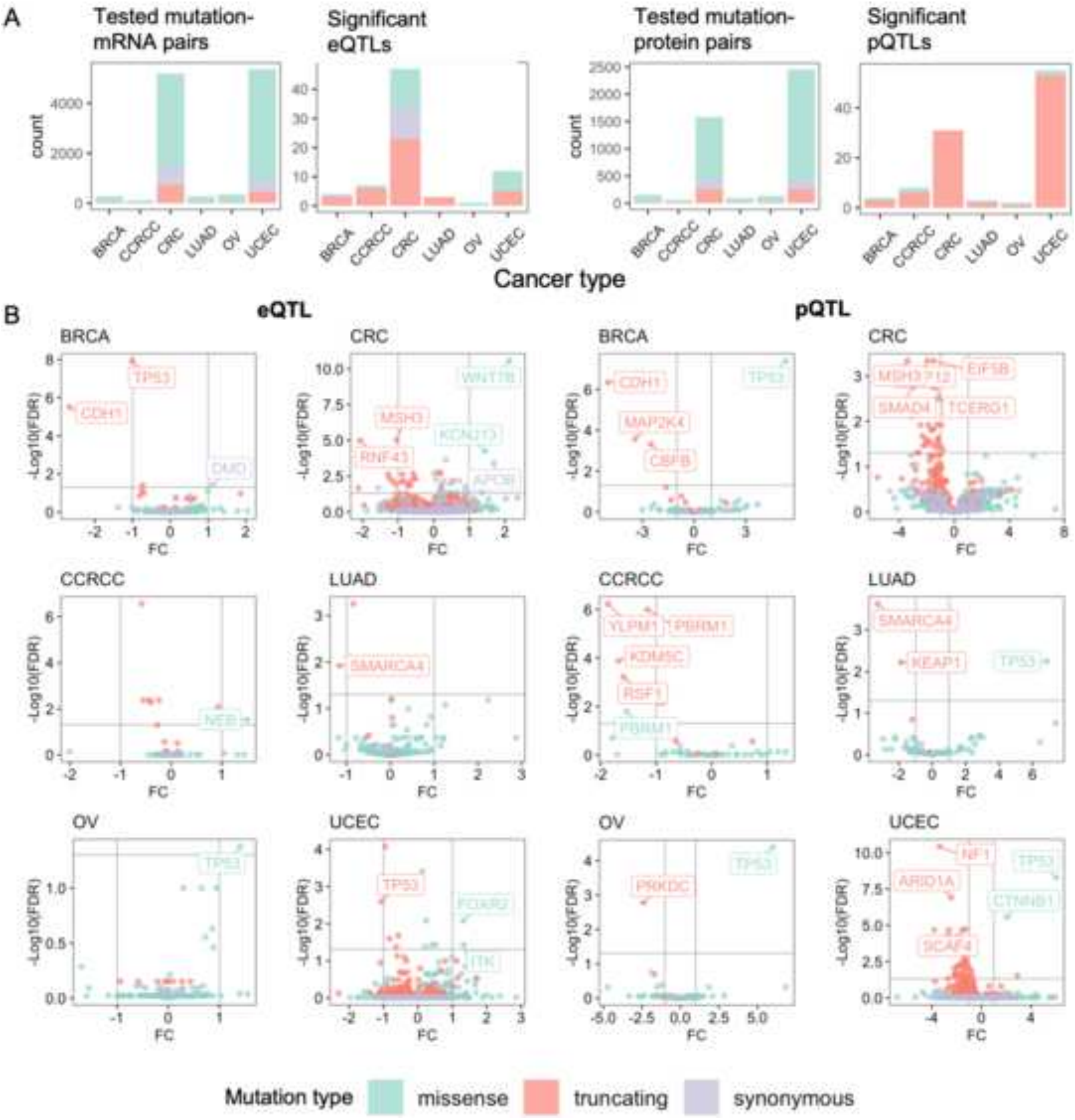

Click here to access/download;Figure;fig3.tiff

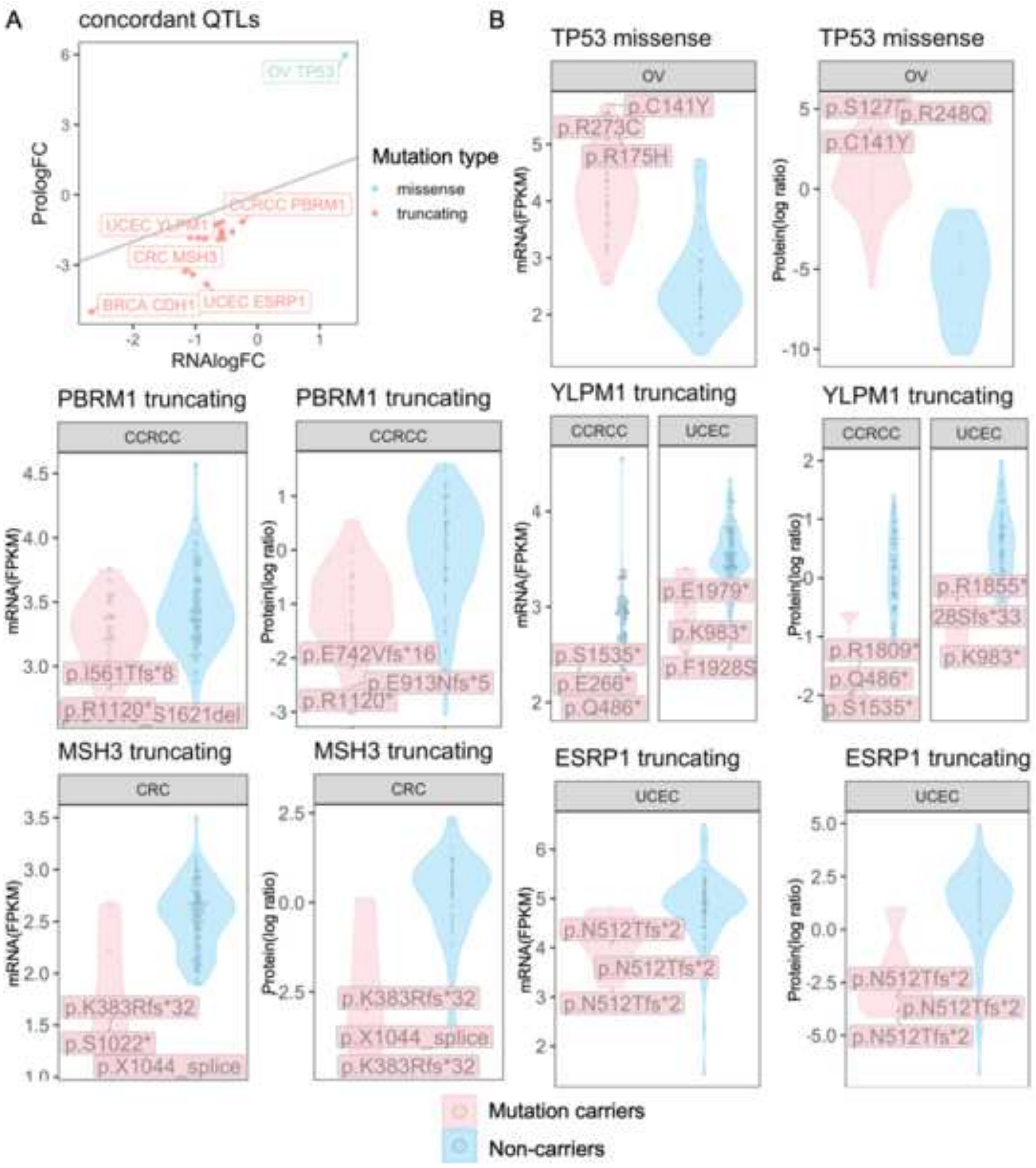

Figure 4 Click here to access/download;Figure;fig4.tiff

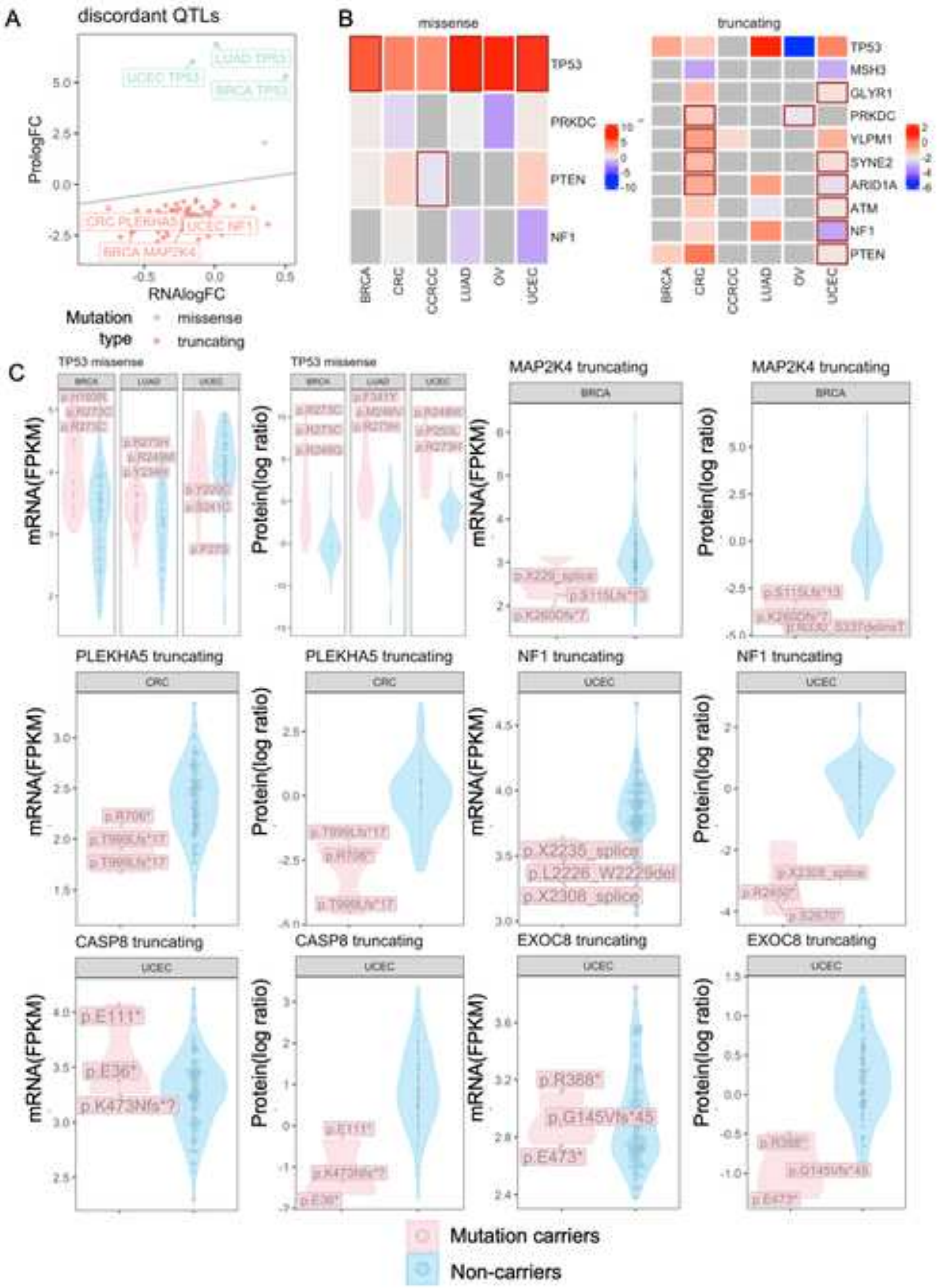

Figure 5

Click here to access/download;Figure;fig5.tiff

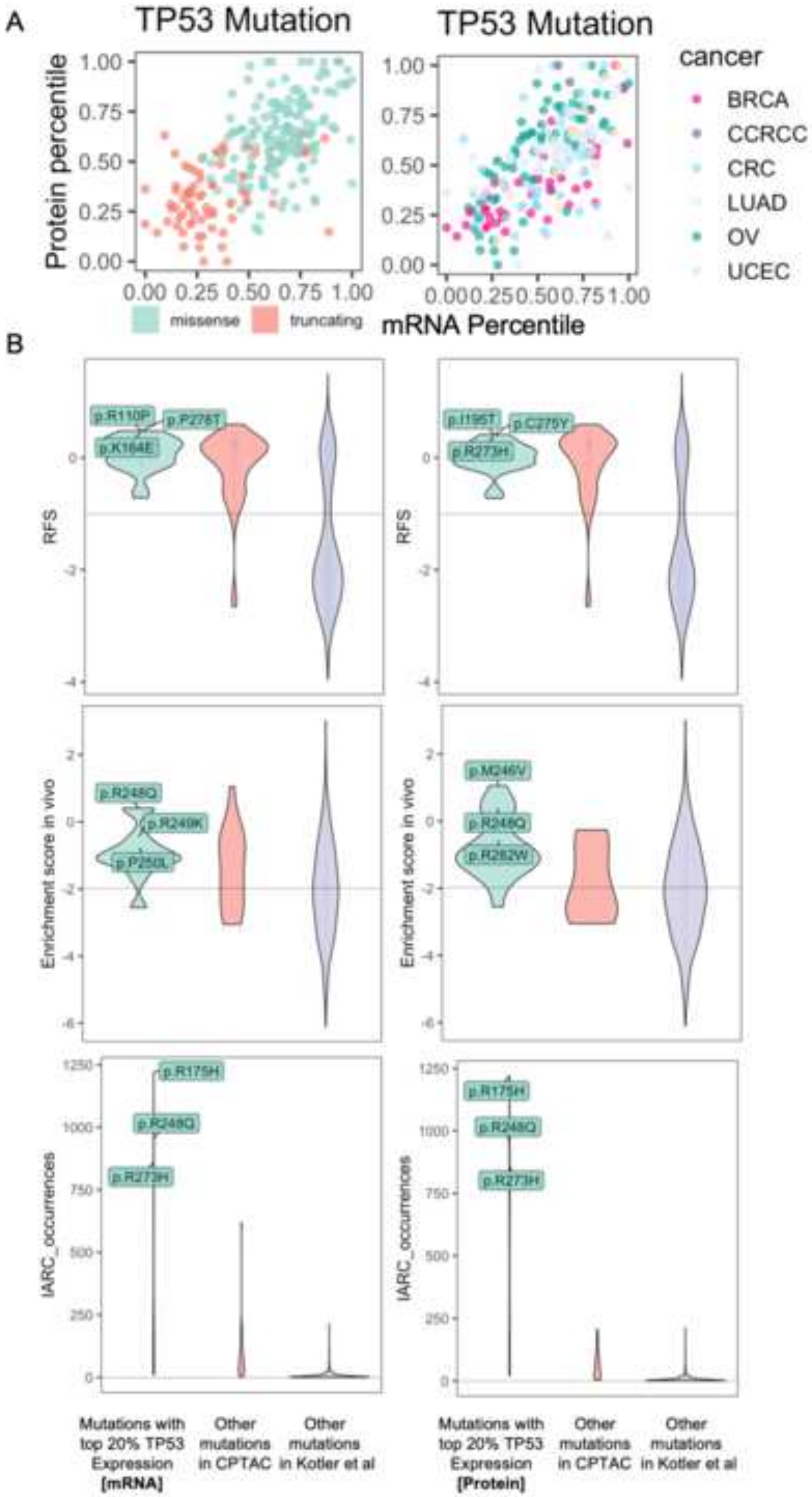

Supplementary Figures

Click here to access/download
Supplementary Material
SuppFigures.pdf

Supplementary Table 1

Click here to access/download
Supplementary Material
TableS1.eQTLs.xlsx

Supplementary Table 2

Click here to access/download
Supplementary Material
TableS2.pQTLs.xlsx

Supplementary Table 3

Click here to access/download
Supplementary Material
TableS3.concordant_e.pQTLs.xlsx

Supplementary Table 4

Click here to access/download
Supplementary Material
TableS4.significant_spsQTLs.xlsx

Supplementary Table 5

Click here to access/download
Supplementary Material
TableS5.DEP_pairedTN_stats.xlsx

Supplementary Table 6

Click here to access/download
Supplementary Material
TableS6.TP53_mutation_test_statistics.xlsx

Supplementary Table 7

Click here to access/download
Supplementary Material
TableS7.CorCoefs.ConcordantAndDiscordant.xlsx

Personal Cover
Click here to access/download;Personal Cover;pQTL_CoverLetter_R2.docx

Icahn School of Medicine at Mount Sinai

Kuan-lin Huang, PhD
Assistant Professor, Department of Genetics and Genomic Sciences
Institute of Genomics and Multiscale Biology
Icahn School of Medicine at Mount Sinai

1399 Park Avenue (Room 4-420C)
Box 1498
New York, NY 10029
Phone: (212) 824-6134
Email: kuan-lin.huang@mssm.edu
Web: ComputationalOmicsLab.org

Nov 21th 2024

Hans Zauner
Editor, GigaScience

Dear Dr. Zauner,

We would like to re-submit our manuscript titled *"Mutation Impact on mRNA Versus Protein Expression across Human Cancers"* (GIGA-D-24-00168), addressing all the editorial comments:

1) Please include a citation to your new GigaDB dataset (including the DOI link) to your reference list, and cite this in the data availability section. Completed.

2) Please structure your abstract ("Background - Results - Conclusions") Completed.

3) Please submit the manuscript text without embedded figures. Please upload the figures separately in Editorial Manager (one file per figure in good resolution - please refer to the formatting instructions on our homepage). Completed.

4) Please move URLs to the bibliography and cite them by reference number, rather than including them in the text directly (e.g. line 562, 570, 571). (We treat internet sources as citable objects). Completed.

5) Please rename the "code availability" section as "Availability of supporting source code and requirements" and use this tabular format: Completed.

6) For reference numbers in the text, please use square brackets (e.g. [1]) instead of superscript numbers. Completed.

7) Please also note the reviewer's comment below regarding the equation - although it looks fine in the Word document I'm looking at now, I believe this was just a conversion problem in

Icahn School of Medicine at Mount Sinai

Kuan-lin Huang, PhD
Assistant Professor, Department of Genetics and Genomic Sciences
Institute of Genomics and Multiscale Biology
Icahn School of Medicine at Mount Sinai

1399 Park Avenue (Room 4-420C)
Box 1498
New York, NY 10029
Phone: (212) 824-6134
Email: kuan-lin.huang@mssm.edu
Web: ComputationalOmicsLab.org

the PDF. Yes it looked fine on our end, attached also the PDF we converted ourselves which looked fine.

8) Please also ensure that your revised manuscript conforms to the journal style, which can be found in the Instructions for Authors on the journal homepage Completed.

Sincerely and on behalf of the team,

Kuan-lin Huang, Ph.D.
Associate Professor of Genetics and Genomic Sciences & Artificial Intelligence and Human Health
Icahn School of Medicine at Mount Sinai
New York, NY 10029
