# Supplementary material for: Mutation impact on mRNA versus protein expression across human cancers
Source: Gigascience. 2025 Jan 6;14:giae113. doi: 10.1093/gigascience/giae113 (PMC11702362; doi:10.1093/gigascience/giae113)
Supplement: giae113_Response_to_Reviewer_Comments_Original_Submission [file giae113_response_to_reviewer_comments_original_submission.pdf]

Author's Response To Reviewer Comments

Authors: We would like to express our gratitude to the editor and the reviewers for the valuable feedback on our manuscript titled “Mutation Impact on mRNA Versus Protein Expression across Human Cancers” (GIGA-D-24-00168). We have carefully considered the comments, particularly regarding the correlation between mRNA and protein expression, and have conducted additional analyses/edits to address each of the concerns listed by reviewers. We are pleased to submit a revised version of the manuscript for your consideration. Below is a detailed response to all reviewers’ comments:

Reviewer #1: Despite the fact that it is already well known that proteomics is important and provides a unique angle to studying cancer, this paper contributes to such knowledge from an interesting angle with the use of published data. The paper can benefit from having further descriptions on the metrics used to measure performance, and should discuss more thoroughly alternative metrics and shortcomings of the current ones. Figures should be better prepared (e.g. Figure 1 can be enlarged or table extracted; Figure 3 Legend is truncated;)

Authors: We thank Reviewer #1 for applauding our novel approach and the feedback. We have expanded the Methods section to provide a more comprehensive description of the statistical metrics used,
“spsQTL identification

We combined two complementary statistical methods to identify spsQTLs. In the first method adopted from Battle et al.4, we compared the following two nested linear models using likelihood ratio test (LRT) with the “anova” function in R:

$$p = \mu + \beta_0 g + \beta_1 r$$

$$p = \mu + \beta_2 r$$

where g is the genotype, r represents RNA level, and p is the protein level. By comparing these models using LRT and filtering results with an FDR less than 0.05, we identified candidate spsQTLs where the genotype (mutation) has a disproportionate impact on protein abundance independent of mRNA expression.

In the second method adopted from Mirauta et al.22, we selected QTLs where the spQTL FDR was less than 0.05 but the corresponding seQTL FDR was greater than 0.05 as candidate spsQTLs, to specifically identify mutations that affect protein levels without influencing mRNA. We then overlapped these two lists of candidate spsQTLs obtained from two complementary methods to identify the final list of spsQTLs for downstream analyses.”

We also added more discussions of alternative approaches and the limitations of our current methods in Discussion, “This study has several limitations… … Fourth, our regression models assumes a linear relationship between mutations (one gene at a time), confounders, and expression, which may not capture more complex, nonlinear effects of mutations on multiple mRNA or protein expression. Future studies could explore non-linear regression models or neural network approaches to better account for these effects. Fifth, we employed two complementary methods to confidently identify spsQTLs that represent true protein-specific regulatory events. However, the reliance on FDR thresholds could still limit the detection of spsQTLs with subtle effects. Alternative approaches, such as Bayesian models that account for prior biological knowledge or hierarchical modeling, could be considered in future analyses to improve the specificity of spsQTL detection. Additionally, while our method focuses on cis-acting mutations, potential trans-acting effects could be missed, a limitation that should be explored in larger datasets or by incorporating network-based analyses.”

We also have revised the figures as suggested. Figure 1 has been enlarged for clarity, and the legend for Figure 3 has been corrected.

Reviewer #2: The manuscript "Mutation Impact on mRNA Versus Protein Expression across Human Cancers" investigates how somatic mutations affect mRNA and protein expression using data from 953 cancer cases across six types. The study identifies that 47.2% of mutations impacting mRNA levels (seQTLs) also affect protein levels, validating their broader impact. A novel statistical method uncovers 83 protein-specific QTLs (spsQTLs), primarily truncating mutations, significantly affecting protein abundance. Functional validation confirms TP53 missense mutations with high protein levels are functional. However, my main concern is the relationship between mRNA expression and protein expression. The low correlation between these two levels may undermine the analysis, suggesting different regulatory mechanisms. If low correlation is observed, the overlap between seQTL and spQTL may lack biological significance. Also, truncating mutations reducing protein expression seems straightforward, but this does not fully address the complex regulation mechanisms. Therefore, I suggest that the authors first compare the correlation between mRNA and protein expression and select cancer types that show high correlation for subsequent analyses. This approach would provide a more robust biological foundation for the study.

Authors: We greatly appreciate Reviewer #2's insightful comments on the low correlation between mRNA and protein expression and their suggestion to focus on cancer types with higher correlation for further analyses. We like to highlight that the low/moderate mRNA-protein correlation is one of the main motivations for our analyses, whereby mutations found to have mRNA effects (more known) may differ from those showing protein expression impacts (less studied). Genomics or eQTL studies in the field often neglect these potential discrepancies in their assumption.

The added analyses and discussion are added to the main text,
"One possible source of spsQTLs is the imperfect correlation between mRNA and protein expression in the affected genes. Additional statistical analyses revealed that this mRNA-protein correlations range widely across genes and cancer types (Figure S5). While genes harboring spsQTLs have lower mRNA-protein correlations in general than genes with concordant eQTL and pQTL, this is not the case for several discordant genes, including MAP2K4 in BRCA and PBRM1 in CCRCC (Table S7). Based on the number of mutations and genes identified, CRC and UCEC reached statistically significant differences between concordant and all other expressed genes (Wilcoxon rank-sum tests, $p = 0.0056$ and $p = 0.022$, respectively); in CRC, mRNA-protein correlations also showed significant differences between discordant and all other expressed genes ($p = 0.013$ and $p = 0.29$, respectively); other cancer types likely did not reach statistical significance likely due to sufficient mutations identified. The imperfect correspondence between gene mRNA-protein correlations and mutation impacts further stresses the need to analyze and consider protein-specific impacts of mutations. Table S7 provides complete mRNA-protein correlation data for all concordant/discordant eQTL/pQTLs in their respective cancer type for in-depth examination."

As the reviewer also pointed out, truncating mutations that reduce protein expression (likely through NMD) seem straightforward but may not fully capture complex regulatory mechanisms. To clarify this, we had added to our discussion other potential post-transcriptional processes, including the role of translation efficiency and context-specific regulatory factors, that may explain the observed discordant effects between mRNA and protein levels,
"This study has several limitations. First, our findings do not distinguish between several potential mechanisms that could lead to discordant effects of mutations on gene and protein expression. One possibility is that the mutation affects the efficiency of translation, leading to changes in protein levels that are not reflected in mRNA levels. For example, accumulating evidence in recent years suggests that NMD is closely tied to the termination of translation23, which may explain instances where some truncations afford much stronger associations with protein levels in our findings. But, in many cases, the mechanisms of how mutations may affect protein abundance may be context- and gene-specific and remain to be elucidated. For example, certain mutations may influence the binding of RNA binding proteins and the efficiency of translation, whereas others may alter post-translational modifications, such as phosphorylation or ubiquitination, which can impact protein stability or degradation without affecting transcription or translation rates."
