# Supplementary material for: Mutation impact on mRNA versus protein expression across human cancers
Source: Gigascience. 2025 Jan 6;14:giae113. doi: 10.1093/gigascience/giae113 (PMC11702362; doi:10.1093/gigascience/giae113)
Supplement: giae113_Response_to_Reviewer_Comments_Revision_1 [file giae113_response_to_reviewer_comments_revision_1.pdf]

Author's Response To Reviewer Comments

We would like to re-submit our manuscript titled “Mutation Impact on mRNA Versus Protein Expression across Human Cancers” (GIGA-D-24-00168), addressing all the editorial comments:

1) Please include a citation to your new GigaDB dataset (including the DOI link) to your reference list, and cite this in the data availability section. Completed.

2) Please structure your abstract ("Background - Results - Conclusions") Completed.

3) Please submit the manuscript text without embedded figures. Please upload the figures separately in Editorial Manager (one file per figure in good resolution - please refer to the formatting instructions on our homepage). Completed.

4) Please move URLs to the bibliography and cite them by reference number, rather than including them in the text directly (e.g. line 562, 570, 571). (We treat internet sources as citable objects). Completed.

5) Please rename the "code availability" section as "Availability of supporting source code and requirements" and use this tabular format: Completed.

6) For reference numbers in the text, please use square brackets (e.g. [1]) instead of superscript numbers. Completed.

7) Please also note the reviewer's comment below regarding the equation - although it looks fine in the Word document I'm looking at now, I believe this was just a conversion problem in the PDF. Yes it looked fine on our end, attached also the PDF we converted ourselves which looked fine.

8) Please also ensure that your revised manuscript conforms to the journal style, which can be found in the Instructions for Authors on the journal homepage Completed.

Sincerely and on behalf of the team,

Kuan-lin Huang, Ph.D.
Associate Professor of Genetics and Genomic Sciences & Artificial Intelligence and Human Health
